# Tcf4 regulates secretory cell fate decisions in the small intestine and colon tumors: insights from transcriptomic, histological, and microbiome analyses

**DOI:** 10.1186/s13287-025-04280-y

**Published:** 2025-04-12

**Authors:** Lucie Janeckova, Monika Stastna, Dusan Hrckulak, Linda Berkova, Jan Kubovciak, Jakub Onhajzer, Vitezslav Kriz, Stela Dostalikova, Tereza Mullerova, Katerina Vecerkova, Marketa Tenglerova, Stepan Coufal, Klara Kostovcikova, Richard S. Blumberg, Dominik Filipp, Konrad Basler, Tomas Valenta, Michal Kolar, Vladimir Korinek

**Affiliations:** 1 Laboratory of Cell and Developmental Biology, Institute of Molecular Genetics, Czech Academy of Sciences, Videnska 1083, Prague 4, 142 20 Czech Republic; 2 Laboratory of Genomics and Bioinformatics, Institute of Molecular Genetics, Czech Academy of Sciences, Prague, Czech Republic; 3 Laboratory of Immunology, Institute of Molecular Genetics, Czech Academy of Sciences, Prague, Czech Republic; 4 Laboratory of Cellular and Molecular Immunology, Institute of Microbiology, Czech Academy of Sciences, Prague, Czech Republic; 5 Gastroenterology Division, Brigham and Women’s Hospital, Boston, USA; 6 Department of Molecular Life Sciences, University of Zurich, Zurich, Switzerland

**Keywords:** Antimicrobial peptides, Paneth cells, Intestinal cell lineage, Colorectal cancer, Intestinal crypt, Single-cell transcriptomics

## Abstract

**Background:**

The canonical Wnt signaling pathway controls the continuous renewal of the intestinal epithelium and the specification of epithelial cell lineages. Tcf4, a nuclear mediator of Wnt signaling, is essential for the differentiation and maintenance of Paneth cells in the small intestine. Its deficiency is associated with reduced expression of key α-defensins, highlighting its role in host-microbe interactions. However, the exact function of Tcf4 in specifying the secretory lineage and its contribution to antimicrobial peptide production remain incompletely understood. Remarkably, α-defensin expression has also been detected in human colon adenomas, where aberrant Wnt signaling is a hallmark. This raises important questions: What is the role of these Paneth-like cells in tumor biology, and how does Tcf4 influence their identity and function?

**Methods:**

We investigated cell specification in small intestinal crypts and colon tumors using conditional *Tcf7l2* deletion, cell type-specific Cre recombinases, and reporter alleles in mice. Transcriptomic (single-cell and bulk RNA sequencing) and histological analyses were performed and complemented by microbiome profiling, antibiotic treatment, and intestinal organoids to functionally validate the main findings.

**Results:**

The inactivation of Tcf4 depletes Paneth cells and antimicrobial peptides, disrupting the gut microbiota balance. In secretory progenitors, loss of Tcf4 shifts differentiation toward goblet cells. In the small intestine, alternative secretory progenitors produce Wnt ligands to support stem cells and epithelial renewal in the absence of Paneth cells. In colon tumors, Paneth-like cells form a tumor cell population, express Wnt ligands, and require Tcf4 for their identity. Loss of Tcf4 redirects their differentiation toward goblet cells.

**Conclusions:**

Tcf4 controls the balance between Paneth and goblet cells and is essential for antimicrobial peptide production in the small intestine. In colon adenomas, Paneth-like tumor cells drive antimicrobial gene expression and provide Wnt3 ligands, which may have implications for cancer therapy.

**Graphic Abstract:**

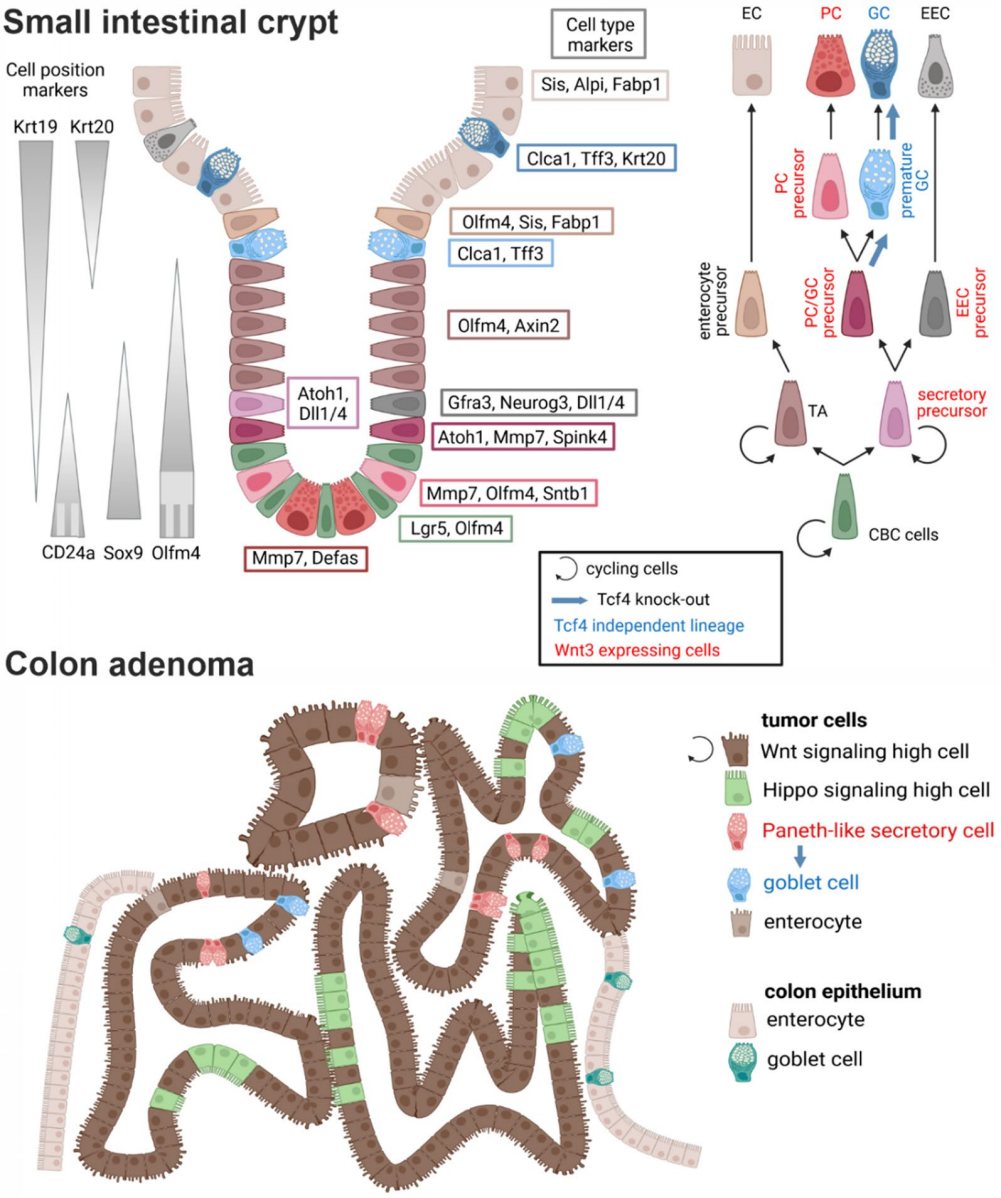

**Supplementary Information:**

The online version contains supplementary material available at 10.1186/s13287-025-04280-y.

## Introduction

The intestinal epithelium undergoes continuous renewal, driven by intestinal stem cells (ISCs) at the crypt base. These ISCs generate progenitor cells that differentiate as they migrate upwards [1]. The cell fate decision is controlled by the opposing activities of Wnt and Notch signaling pathways: the Notch pathway promotes differentiation into absorptive enterocytes, while its absence directs cells toward the secretory lineage [2]. Goblet cells, the most abundant secretory cells, produce mucin 2 (Muc2), an important component of the mucus layer [3]. Paneth cells, located at the crypt base, are distinguished from goblet cells by their active Wnt signaling and their role in maintaining the ISC niche through antimicrobial defense and secretion of Wnt and Notch ligands [4]. In the colon, deep crypt secretory (DCS) cells share features with Paneth cells but do not produce Wnt ligands. Instead, they contribute to the host defense by producing peptides and mucins [5,6].

The Paneth cell function depends on T cell factor 4 (Tcf4), encoded by the *Tcf7l2* gene [7,8]. As a key mediator of Wnt signaling, Tcf4 is essential for maintaining ISC pluripotency [9]. The complete knockout of *Tcf7l2* in mice leads to perinatal lethality, primarily due to the lack of proliferative compartments in the small intestine [9]. Tissue-specific deletion of Tcf4 in the intestinal epithelium of adult mice results in the loss of proliferating cells, thereby blocking the self-renewal capacity of the small intestinal epithelium [8,10].

In this study, we investigated the effects of Tcf4 loss on cell fate in the lower crypt epithelium of the mouse small intestine. To prevent premature death of the experimental animals, we used conditional *Tcf7l2* gene alleles in combination with the *Defa6-iCre* driver, which is active in Paneth cells and their precursors. Reporter mice (*Rosa26-tdTomato* and *Mki67*^*RFP*^) were used to track the fate of Tcf4 wild-type (WT) and Tcf4-deficient cells. To uncover changes in secretory cell differentiation, we performed a detailed analysis using single-cell and bulk RNA sequencing in combination with immunohistochemical staining of specific cell markers.

In addition, Paneth and goblet cells play a key role in maintaining the barrier between host tissue and the gut microbiota. Mutations in *TCF7L2* are associated with Crohn's disease in the human ileum, as Paneth cell depletion leads to decreased levels of antimicrobial peptides α-defensins 5 and 6 (DEFA5/6), resulting in chronic inflammation of the intestinal mucosa [7]. We leveraged the altered cellular composition of small intestinal crypts following Tcf4 loss as a model to study its impact on microbiome composition. Since Paneth cells are an important source of Wnt3 ligand for crypt cells in the intestinal epithelium [11], we also investigated the effects of Tcf4 loss on their development and function in intestinal organoids [12]. These organoid cultures lack intestinal mesenchymal cells, which serve as an additional source of Wnt ligands [13,14]. Thus, the loss of proliferative capacity or phenotypic changes in organoid cells served as a functional assay.

Physiological Wnt signaling is essential for cell proliferation and epithelial renewal, but its dysregulation drives gastrointestinal tumorigenesis [15,16]. Colorectal cancer (CRC) develops through cumulative genetic mutations, with *APC* inactivation being one of the earliest and best-documented events. Loss of *APC* leads to β-catenin accumulation and aberrant Wnt pathway activation, independent of external Wnt signals [17]. In our previous study, we observed strong activation of Paneth cell-specific gene expression in the colonic epithelium of mice within a few days after conditional *Apc* inactivation [18]. We used this finding to test whether the *Defa6-iCre* driver can specifically mark nascent colorectal tumor cells. This was confirmed, enabling us to use this mouse line to inactivate *Tcf7l2* in tumor cells and study the effects of Tcf4 loss on their development. By combining *Defa6-iCre* with a reporter allele, we were able to track developmental trajectories exclusively in transformed colon cells, overcoming a major limitation of conventional mouse models of tumorigenesis, in which analyzed cells often consist of a mixture of healthy and transformed epithelial cells.

Finally, in another study, we showed that Tcf4 is essential for both proliferation and tumor formation in the small and large intestines of mice [10]. However, in the context of human CRC, the TCF4 role remains unclear, particularly because inactivating mutations in the *TCF7L2* gene are relatively common in advanced stages of human colorectal cancer. These findings suggest that *TCF7L2* may function as a tumor suppressor, at least in human CRC. Using conditional alleles of *Tcf7l2* and *Apc* in combination with *Defa6-iCre*, we were able to analyze the effects of Tcf4 loss on a subpopulation of cells within the developing tumor.

## Materials and methods

The work has been reported in line with the ARRIVE guidelines 2.0. No human cells or tissues were used in this research. All results reported in this study were obtained by analyzing intestinal tissue and microbiota from mice with a cell-specific conditional knockout of *Tcf7l2* in comparison to their WT littermates (at least four animals per group). The target cells were identified using reporter alleles. For the analysis of intestinal adenomas, we used mice with multiple intestinal neoplasia (*Apc*^+*/Min*^) [19]. As an alternative model for intestinal tumorigenesis, we used the mutagen azoxymethane (AOM) in combination with dextran sulfate sodium (DSS)-induced colitis [20].

### Experimental mice

*Apc*^+*/Min*^ mice [19], *Mki67*^*RFP*^ mice [21], *Pdgfra-CreER*^*T2*^ mice [22], *ROSA26-tdTomato* mice [*B6;129S6-Gt(ROSA)26*^*Sortm14(CAG−tdTomato)Hze*^*/J*] [23], *ROSA26-CreER*^*T*2^ mice [*B6.129-Gt(ROSA)26*^*Sortm1(cre/ERT2)Tyj*^*/J*] [24] and ROSA-DTA mice [*B6.129P2-Gt(ROSA)26*^*Sortm1(DTA)Lky*^*/J*] [25] were purchased from The Jackson Laboratory (Bar Harbor, ME, US). *Villin-CreER*^*T2*^ mice [26] were kindly provided by S. Robine (Institut Curie, Centre de Recherche, Paris, France). *Tcf7l2f*^*lox/flox*^ mice were obtained from the European Conditional Mouse Mutagenesis Program (EUCOMM; Wellcome Trust Sanger Institute) and have been previously described [10]. *Defa6-iCre* [27] and *Wls*^*flox/flox*^ [13] mice have also been described previously. Animals were maintained under specific pathogen-free (SPF) conditions in enriched environment and genotyped according to the provider’s protocols or published protocols. No animal anesthesia was used in this research. Total number of 265 animals was used for these experiments.

### Cre-mediated gene recombination

Adult mice (8–22 weeks old) producing CreER^T2^ were administered 250 mg/kg tamoxifen (Sigma-Aldrich, St. Louis, MO, USA; 100 mg/mL stock solution in ethanol). The tamoxifen solution was mixed with mineral oil (Sigma-Aldrich) prior to a single administration by gavage. Mice were sacrificed by cervical dislocation at the time points indicated in each experiment after a single administration of the tamoxifen solution. Colon tumors were isolated from 13–19 week old Apc-deficient mice.

### Colitis and tumor induction

Inflammatory damage to the colon was induced by 2% (w/v) DSS (MP Biomedicals, Irvine, CA, USA; MW36–50 kDa) in drinking water for 5 days. After discontinuation of DSS on day 8 (recovery period after colitis), the colons were removed and analyzed. To ensure inflammation-induced tumorigenesis, mice were injected i.p. with AOM (10 mg/kg; Sigma-Aldrich) 7 days prior to DSS administration. The mice were sacrificed 3 weeks after discontinuation of DSS. To ensure sufficient numbers of tdTomato^+^ tumor cells for organoid seeding, we used prolonged AOM/DSS treatment in Apc-deficient mice – 7 days after a single injection of AOM (10 mg/kg), 1% (w /v) DSS was administered for 5 days, which was repeated three times at 14-day intervals. Subsequently, the tumors were processed.

### Antibiotic treatment

To eliminate the gut microbiome in *Tcf7l2*^*flox/flox*^*/Villin-CreER*^*T2*^ mice, vancomycin hydrochloride (PHR1732, Sigma-Aldrich; working concentration 500 mg/L) was added to the drinking water 7 days before tamoxifen administration in the indicated experiments; the vancomycin-containing water was changed every 7 days.

### Isolation of intestinal tissues and epithelial cells

For immunohistochemical staining, the intestines were dissected, washed in phosphate-buffered saline (PBS), fixed in 10% buffered formaldehyde solution (Sigma-Aldrich), embedded in paraffin, sectioned and stained. For organoid cultures and gene expression analysis, the intestinal crypts were isolated from the proximal jejunum of the respective mice. The intestinal tube was cut open lengthwise and the villi were carefully scraped off with a coverslip. The tissue was washed in PBS and incubated in 5 mM EDTA solution in PBS (pH 8; Merck Millipore, Burlington, MA, USA) at 4 °C for 30 min. The solution was then gently shaken to obtain a suspension of crypts. The crypt suspension was sieved through a 70-μm sieve (Corning, Corning, NY, USA) and centrifuged at 300 × g at 4 °C for 5 min. For tissue isolation for flow cytometry, the villi were not removed and the whole epithelium obtained after incubation with 5 mM EDTA was used. The epithelium was centrifuged at 300 × g for 5 min at 4 °C and resuspended in cleavage medium (serum-free Dulbecco’s Modified Eagle’s Medium; DMEM) with dispase (Thermo Fisher Scientific, Waltham, MA, USA; stock solution 100 mg/ml, diluted 1:300) and DNase I (Thermo Fisher Scientific; working concentration 1 U/ml). The epithelium was incubated 3 × 5 min at 37 °C on a rotating platform (800 × RPM, 5 min, 37 °C). Alternatively, colon tumors were harvested directly from the epithelium and cut into small pieces in a cleavage medium with added collagenase type II (C6885, Sigma-Aldrich; working concentration 1 µg/ml). The tumors were incubated 3 × 10 min at 37 °C on a rotating platform (800 × RPM, 5 min, 37 °C). After each incubation, the tissues were pipetted up and down with a cut tip, and the solution containing the released cells was transferred to DMEM with 10% fetal bovine serum (FCS) to stop cleavage. The collected cells were centrifuged at 300 × g for 5 min at 4 °C and stained.

### Fluorescence-activated cell sorting (FACS)

Epithelial crypt cells from the ileum of *Mki67-RFP Tcf7l2*^*flox/flox*^* Villin-CreER*^*T2*^ mice were stained with Pacific Blue™ (PB) conjugated anti-CD45 antibody (#103,126, BioLegend, San Diego, CA, USA; dilution 1:200), PB-conjugated anti-CD31 (#102,422, BioLegend; 1:200), fluorescein (FITC)-conjugated anti-EpCAM antibody (#11–5791-82, Thermo Fisher Scientific; 1:400) and allophycocyanin (APC)-conjugated anti CD-24 antibody (#17–0242-82, Thermo Fisher Scientific; 1:400) for 20 min at 4 °C; shortly before sorting, Hoechst 33,258 (Merck Millipore) was added to the cell suspension. Cells were sorted by forward scatter (FSC), side scatter (SSC), and negative staining for Hoechst and PB. EpCAM^+^ (epithelial) cells were further sorted for RFP and CD24 expression to obtain EpCAM^+^ RFP^+^ CD24^high^ cells, i.e., proliferating epithelial crypt cells. The same staining and sorting strategy was used for *Tcf7l2*^*flox/flox*^*/ROSA26-tdTomato/Defa6-iCre* mice; the red fluorescence of tdTomato was used to distinguish recombined cells. Cell sorting was performed using the Influx Cell Sorter (BD Biosciences, San Jose, CA, USA). Cell suspension from each animal was sorted separately.

### Organoid cultures

Epithelial crypts from resected mouse intestines were embedded in Matrigel (Corning) and cultured as previously described [28]. Complete organoid culture medium (ENR): Advanced DMEM/F12 culture medium (Thermo Fisher Scientific) was supplemented with GlutaMax (Thermo Fisher Scientific), 10 mM HEPES (1 M stock, Thermo Fisher Scientific), penicillin/streptomycin (Thermo Fisher Scientific), B27 Supplement (Thermo Fisher Scientific), N2 Supplement (Thermo Fisher Scientific), 1.25 mM N-acetylcysteine (Merck Millipore), 50 μg/ml recombinant mouse epidermal growth factor (EGF; Thermo Fisher Scientific), 2 μl/ml Primocin® (InvivoGen, Toulouse, France) and conditioned culture medium (CM) of mNoggin-Fc [29,30] and R-Spondin 1 (Rspo1) [29] at a final concentration of 10% CM each. Cells producing the indicated secreted proteins were kindly provided by H. Clevers (Hubrecht Laboratory, Utrecht, Netherlands) and K. Cuo (Stanford University, USA), respectively. If required, an additional 0.5 nM Wnt surrogate Fc fusion protein (WntSur; U-Protein Express BV, Utrecht, The Netherlands) was added to the culture medium. Using FACS-sorted tdTomato^+^ cells, 10,000 to 20,000 cells were collected in ENR medium containing the Rho-associated protein kinases (Rock) inhibitor Y-27632 (2.5 mM, Sigma-Aldrich) and 5% Matrigel (Corning). Cells were centrifuged at 300 × g for 5 min at 4 °C, embedded in Matrigel and cultured in ENR medium containing WntSur and Rock inhibitor Y-27632 (2.5 mM) for at least 5 days. After the first passage, WntSur and Y-27632 were removed from the culture medium. Cre-mediated recombination in the organoids was induced by adding 4-hydroxytamoxifen (4-OHT) (Sigma-Aldrich; final concentration 2 µM, 1 mM stock solution was prepared in ethanol) to the culture media. Organoids in culture were imaged using a Leica DMI8 wide-field inverted microscope. Organoid cultures were generated from two biological replicates.

### Immunohistochemical staining

A detailed protocol of immunohistochemical staining of paraffin-embedded tissues [18] and organoids [10] has already been described. At least four biological replicates were always used for histological analysis. Primary antibodies: anti-Alpi (rabbit polyclonal, PA5-22,210, Thermo Fisher Scientific); anti-ChgA (rabbit polyclonal, ab15160, Abcam, Cambridge, UK); anti-cleaved Casp3 (rabbit monoclonal, #9664, Cell Signaling Technology, Danvers, MA, USA); anti-Krt20 (mouse monoclonal, M7019, Agilent Dako, Santa Clara, CA, USA); anti-Lysozyme (rabbit polyclonal, A0099, Agilent Dako); anti-Muc2 (rabbit polyclonal, sc-15334, Santa Cruz Biotechnology, Dallas, TX, USA); anti-Olfm4 (rabbit monoclonal, #39,141, Cell Signaling Technology); anti-PCNA (rabbit polyclonal, ab18197, Abcam); anti-PCNA (mouse monoclonal, ab29, Abcam); anti-Pdgfra (goat polyclonal, AF1062, R&D Systems, Minneapolis, MN, USA); anti-Reg3b (sheep polyclonal, AF5110, R&D Systems); anti-RFP (rabbit polyclonal, 600–401-379, Rockland, Pottstown, PA, USA); anti-RFP (mouse monoclonal, MA5-15,257, Thermo Fisher Scientific); anti-Tacstd2 (rabbit monoclonal, ab214488, Abcam); anti-Tcf4 (rabbit monoclonal, #2569, Cell Signaling Technology); anti-Tcf4 (rabbit monoclonal, MA5-35,295, Thermo Fisher Scientific). Secondary antibodies (all from Thermo Fisher Scientific): goat anti-rabbit IgG (H + L) Alexa Fluor™ 488 (A11034); goat anti-rabbit IgG (H + L) Alexa Fluor™ Plus 594 (A32740); goat anti-mouse IgG (H + L) Alexa Fluor™ 488 (A11001); goat anti-mouse IgG (H + L) Alexa Fluor™ 594 (A11005); donkey anti-goat IgG (H + L) Alexa Fluor™ 488 (A11055); donkey anti-sheep IgG (H + L) Alexa Fluor™ 488 (A11015). Cells were counterstained with DAPI nuclear stain (Sigma-Aldrich). Microscopic images were acquired with the Leica Stellaris confocal platform or, in the case of organoids, with the Andor Dragonfly 503 Spinning Disk confocal microscope. The images were processed and analyzed with the FiJi package [31]. Olfm4-positive intestinal crypts were quantified manually. For counting, a single, linear section of the intestine was selected from the 3 × 3 composite image, corresponding to approximately 1700 µm of intestinal length. Olfm4-positive crypts were counted from four biological replicates for the duodenum, jejunum, and ileum. In each intestinal region, three technical replicates were analyzed for each group.

### DNA and RNA isolation and analysis

Total RNA from intestinal epithelial cells (freshly isolated crypts or FACS-isolated samples from at least two biological replicates) was isolated using the RNeasy Micro Kit (Qiagen, Germantown, MD, USA) and reverse transcribed using MAXIMA Reverse Transcriptase (Thermo Fisher Scientific) according to the manufacturer’s protocol. Reverse transcription—quantitative polymerase chain reaction **(**RT-qPCR) was performed in triplicate using the SYBR Green I Master Mix and the LightCycler 480 instrument (Roche Diagnostics, Indianapolis, IN, USA). For simultaneous isolation of genomic DNA from sorted cells, we used the AllPrep DNA/RNA Micro Kit (Qiagen). The presence of the floxed and recombined allele was analyzed by PCR using the EliZyme™ HS Robust Mix (Elisabeth Pharmacon, Prague, Czech Republic). The primers used for PCR and RT-qPCR are listed in Supplementary material 1: Table S1. Isolation and microarray analysis of *Apc*^*cKO/cKO*^*/VillinCreER*^*T2*^ colonic epithelium 2 and 4 days after recombination was described in a previous study [18].

### Bulk RNA sequencing (bulk RNA-seq) and computer analysis

The quantity and quality of the isolated RNA from at least four biological replicates was measured with the NanoDrop ND-2000 (NanoDrop Technologies, Wilmington, DE, USA) and analyzed with the Agilent 2100 Bioanalyzer (Agilent Technologies, Santa Clara, CA, USA). The isolated RNA was processed using the Takara Smarter Stranded Total RNA-seq Kit v2 Pico Input Mammalian according to the manufacturer's instructions. Libraries were sequenced using either the NextSeq 500 or 2000 instrument (both Illumina, CA, USA), with the length set to 75 bases for *Mik67*^*RFP*^*/Tcf7l2*^*flox/flox*^*/Villin-CreER*^*T2*^ and 122 bases for *Tcf7l2*^*flox/flox*^*/ROSA26-tdTomato/Defa6-iCre* libraries. Subsequent processing of the *Mik67*^*RFP*^*/Tcf7l2*^*flox/flox*^*/Villin-CreER*^*T2*^ data was performed using the nf-core/rnaseq version 1.4.2 bioinformatics pipeline [32]. The individual steps included the removal of sequencing adapters and low-quality reads with Trim Galore! (www.bioinformatics.babraham.ac.uk/projects/trim_galore), mapping to the reference genome GRCm38 (Ensembl annotation version 98) [33] with HISAT2 version 2.1.0 [34] and quantification of gene expression with FeatureCounts [35]. The estimated expression per gene served as input for differential expression analysis using the DESeq2 R Bioconductor package [36]. Only genes that were expressed in at least two samples were considered for the test. We compared expression between sample groups based on the *Tcf7l2* conditional knockout (cKO) status of most epithelial cells. Genes that had a minimum absolute log_2_ fold change of 1 (|log_2_ FC|≥ 1) and statistical significance (adjusted P-value < 0.05) between the compared sample groups were considered differentially expressed.

Data from *Tcf7l2*^*flox/flox*^*/ROSA26-tdTomato/Defa6-iCre* libraries were analyzed with the nf-core/rnaseq pipeline [32] version 3.12 using STAR [37] and Salmon [38] to quantify expression per gene using the GRCm39 assembly (Ensembl annotation version 104). Minimal expression (counts) of 10 per gene was used across samples. Differentially expressed genes were identified using a minimum absolute log_2_ fold change of 1 (|log_2_ FC|≥ 1) and statistical significance (adjusted P‐value < 0.1). In addition, the enrichment of KEGG pathways and Gene Ontologies (GO terms) was analyzed using the gene set enrichment analysis (GSEA) method implemented in the ClusterProfiler R Bioconductor package [39]. The initial mapping and analysis of the sequencing data was performed by the Genomics and Bioinformatics Core Facility at the Institute of Molecular Genetics of the Czech Academy of Sciences. For additional GSEA analyses, the Enricher web platform was used [40,41].

### Single-cell RNA sequencing (scRNA-seq) and computer-assisted analyses

For scRNA-seq analysis, cells from two biological replicates were pooled together to eliminate the bias caused by individual animal differences. Barcoded single-cell cDNA libraries were prepared with The Chromium Controller (10X Genomics, Pleasanton, CA, USA) using the Chromium Next Gem Single Cell 30 Kit, v3.1, according to the manufacturer's protocol. The barcoded cDNA was then pooled and sequenced using the NextSeq 500 instrument (Illumina) with an mRNA fragment read length of 130 bases (119 bases for the RFP_WT_GERM sample). We used the 10X Genomics Cell Ranger analysis suite to quantify gene expression per cell based on the GRCm38 Ensembl 98 genome (GRCm39 Ensembl 104 for RFP_WT_GERM). The obtained sequencing data were then analyzed in R Studio with Rx64 3.6.2.Ink (R-tools Technology Inc., Richmond Hill, Canada) using Seurat version 3.1 [42]. Only cells with a number of 200 to 7,000 genes and genes detected in more than three cells were included in the quality control. In the experiment in which proliferating epithelial cells were analyzed, cells with more than 10% mitochondrial (mt) gene content were excluded from the analysis, reflecting the higher metabolic activity and consequently higher proportion of mt genes in the small intestinal epithelium [43]. This filtering resulted in 5,215 cells with 14,308 genes in the *Tcf7l2* WT dataset and 3,859 cells with 14,704 genes in the *Tcf7l2* cKO dataset. In the colon tumor analysis, we found an overall higher proportion of mt genes, which is consistent with previously published bioinformatic analyses of tumor tissues [44]. Due to the increased metabolic activity of the tumor tissue, we excluded cells with more than 25% mitochondrial gene content. This approach resulted in 1,096 cells with 16,715 genes in the whole tumor dataset and 1,406 cells with 14,457 genes in the sorted tumor tdTomato^+^ cell dataset. In both experiments, the datasets were merged, and normalization, scaling and variable gene selection were performed with default settings. Cell clusters were identified using the Louvain approach based on principal component analysis (PCA). Nonlinear dimensionality reduction by Uniform Manifold Approximation and Projection (UMAP) [45] was applied to visualize the low-dimensional embedding of the data and confirm the cluster assignment of the cells.

For the prediction of future gene expression of cells in clusters, we analyzed the scRNA velocity based on the ratio of spliced and unspliced mRNA [46]. First, we used the Python package Velocyto 0.17 to calculate separate expression matrices for spliced and nascent mRNA. In the next step, the velocity vectors per cell were calculated using the workflow of the package scVelo 0.2.4 assuming the standard stochastic model. Finally, the velocities were projected onto the UMAP embedding of cell clusters created with the Seurat toolkit.

### Microbiome analysis and data processing

*Mki67*^*RFP*^*/Tcf7l2*^*flox/flox*^*/Villin-CreER*^*T2*^ mice (7 Cre^+^ and 8 Cre^−^ littermates; mice of the same genotype were kept separate to avoid contamination of the microbiome) were sacrificed 7 days after tamoxifen-induced recombination and the contents were isolated from the ileum of the small intestine. Total DNA was extracted using the ZymoBIOMICS DNA Miniprep Kit (ZYMO Research, Irvine, CA, USA) by repeated bead beating with the FastPrep Homogenizer (MP Biomedicals). The DNA was then quantified using the Qubit dsDNA High Sensitivity Kit (Thermo Fisher Scientific). Samples were processed as technical duplicates to increase the accuracy of the sequencing data. In addition, a ZymoBIOMICS gut microbiome standard (ZYMO Research) was used as a positive control in preparing the sequencing libraries.

The sequencing libraries were prepared using a two-step PCR procedure [47]. The first PCR was performed using Kapa HiFi DNA polymerase (Kapa Biosystems, Wilmington, MA, USA) and primers S-D-Bact-0341-b-S-17 (CCTACGGGGGGNGGCWGCAG) and S-D-Bact-0785-a-A-21 (GACTACHVGGGGTATCTAATCC) targeting the V3 and V4 regions of bacterial 16S. The primers contained inline barcodes at the 5´ end and 10-bp tails that were recognized by the second primer pair. Cycling conditions consisted of initial denaturation (95 °C, 3 min), followed by 28 cycles of denaturation (98 °C, 20 s), annealing (55 °C, 30 s) and extension (72 °C, 30 s), with final extension (72 °C, 5 min). In the second PCR, the unique indices and sequences were added based on the TruSeq adapters. Cycling conditions consisted of initial denaturation (95 °C, 3 min), followed by 12 cycles of denaturation (98 °C, 20 s), annealing (55 °C, 30 s) and extension (72 °C, 30 s) with final extension (72 °C, 5 min). Products were quantified using QIAxcel Advanced Capillary Electrophoresis (Qiagen), and samples within the library were pooled in equal proportions. Libraries were further purified with SPRIselect beads (Beckman Coulter, Brea, CA, USA) and sequenced on the MGI platform at The Genomics Core Facility, CEITEC (Brno, Czech Republic).

Demultiplexing, primer detection and trimming of the sequencing data were performed using Skewer [48]. Reads of low quality (expected error rate per paired-end read > 4) were then eliminated. DADA2 [49] was used to denoise the quality-filtered reads and quantify 16S rRNA Amplicon Sequence Variants (ASVs) in each sample. Chimeric ASVs were detected and eliminated using UCHIME [50] and the Silva database [51]. Taxonomic assignment of non-chimeric ASVs was performed using the Ribosomal Database Project (RDP) classifier with 80% confidence threshold [52] and the latest version of the Silva database [51]. Using Procrustean analysis, we checked the consistency of haplotype composition between identical profiles and retained only the haplotypes that were present in both technical duplicates. We found a high consistency between the technical duplicates. Chloroplasts as well as sequences that could not be assigned to any bacterial strain were considered as food contaminants or sequencing artifacts and excluded from all downstream analyses. The sequences of technical duplicates were pooled for each sample. The ASV abundance matrix (i.e., the number of ASV reads in each sample), the ASV sequences, their taxonomic annotations and phylogeny were merged into a single database together with the metadata of the samples using the phyloseq package [53] in R (R Core Team 2020, Vienna, Austria; http://www.r-project.org/index.html). Taxonomic analysis was performed using the microViz package [54]; relative abundances were calculated. Visualization of microbial composition through the iris plot was performed using the centered log ratio (CLR) transformation of taxa at the genus level. PCA was performed to investigate differences in microbiota composition between groups.

### Statistical analysis

Statistical analyses were performed using R, version 3.6.2. Exploratory data analysis was performed for all parameters. Data are presented as mean +— standard deviation (SD; normally distributed data). Survival probabilities for cancer-specific survival were determined using the Kaplan–Meier method and the log-rank test. At least 4 animals of the same genotype in each group were always used for the quantification of obtained results to ensure significant statistical results. The results of RT-qPCR analysis and immunofluorescence quantification in FiJi were analyzed using a one-way ANOVA test; a p-value < 0.05 was considered significant. The normality distribution of the data was checked for each set of measurements. Randomization was not performed in order to compare groups with different genotypes.

Graphical abstract was created with BioRender.com.

## Results

### Intestinal epithelial cell dynamics and microbiota composition are altered by inactivation of Tcf4 in a conditional mouse model

**Fig. 1:**
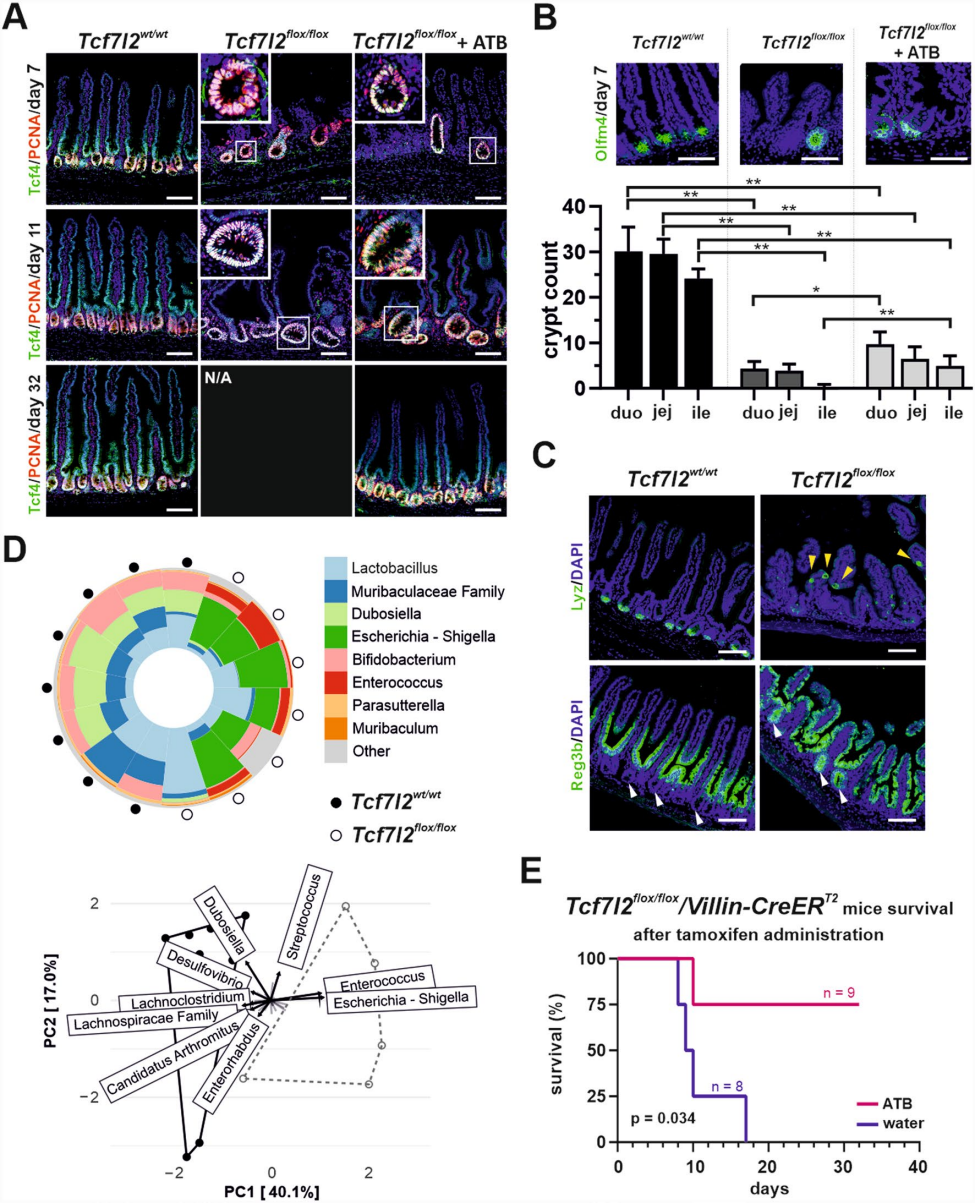
Knockout of transcription factor 7-like 2 (*Tcf7l2*) gene affects intestinal epithelial renewal and microbiota dynamics. **A** Fluorescence micrographs of T-cell factor 4 (Tcf4, green) and proliferating cell nuclear antigen (PCNA, red) localization in the small intestine of wild-type *Tcf7l2*^*wt/wt*^*/VillinCreER*^*T2*^ (*Tcf7l2*^*wt/wt*^) and knockout *Tcf7l2*^*flox/flox*^* /VillinCreER*^*T2*^ (*Tcf7l2*^*flox/flox*^) mice, with or without antibiotic (ATB) treatment, over time after tamoxifen administration. Greyscale images are shown in Additional file 8: Supplementary Figure S1. The insets show increased magnification of the framed areas. Scale bar: 50 μm. **B** Quantification of small intestinal crypts with the ability to renew the intestinal epithelium. Samples were stained with an antibody against the stem cell marker olfactomedin 4 (Olfm4; green signal; scale bar: 50 μm). Olfm4-positive crypts were manually counted in the three fields of view in the duodenum (duo), jejunum (jej) and ileum (ile) of the four biological replicates. The statistical significance between the individual groups in the corresponding part of the small intestine was determined using the one-way ANOVA test; *p < 0.05; **p < 0.01. **C** Histological sections show the mislocalization and expression changes of the Paneth cell markers lysozyme (Lyz) and regenerating islet-derived 3 beta (Reg3b) seven days after tamoxifen administration. Yellow arrowheads indicate Lyz-positive cells on the villi; white arrowheads show that the crypt bases in the Tcf4^+^ epithelium are not positive for Reg3b, while they produce Reg3b in the Tcf4-deficient epithelium. Scale bar: 50 μm. **D** Analysis of the microbiome composition in the distal small intestine of *Tcf7l2*^*wt/wt*^ and *Tcf7l2*^*flox/flox*^ mice. The pie chart shows the bacterial distribution in Tcf4-proficient mice (a solid black point on the outer perimeter of the chart) and Tcf4-deficient mice (an empty black ring). The principal component analysis (PCA) below the diagram illustrates the variance between bacterial populations, indicating shifts in the microbiota associated with the *Tcf7l2* status. **E** Kaplan–Meier survival curves for *Tcf7l2* knockout mice treated with either antibiotics or water. Statistical significance (p = 0.034) shows that the antibiotic treatment increases survival. The number of mice used in the experiment is indicated (n), and the graph depicting mouse weight after recombination is shown in Additional file 8: Supplementary Figure S2C

Inactivation of Tcf4 in all small intestinal epithelial cells leads to loss of proliferating cells and subsequent death of experimental animals between 8–16 days after administration of tamoxifen in *Tcf7l2*^*flox/flox*^*/Villin-CreERT2* animals carrying the conditional knockout (cKO) *Tcf7l2* alleles (indicated as *Tcf7l2*^*flox/flox*^). Since the loss of the *Tcf7l2* gene is not complete (100%), proliferating hyperplastic lesions are observed in the small intestine (Fig. 1A,B and Supplementary material 8: Supplementary Fig. S1). The absence of Tcf4 in the epithelium is accompanied by misplaced lysozyme-positive cells (Fig. 1C). We also observed an increased presence of regenerating islet-derived 3 beta (Reg3b), which in a healthy intestine occurs mainly at the interface between the crypts and the intestinal surface. In the Tcf4-deficient intestine, Reg3b was expressed almost ubiquitously, including the remaining crypts that regenerate the epithelium (Fig. 1C). The expression of Reg3b is regulated by the intestinal microbiota and serves to protect the intestinal epithelium from inflammation [55,56]. We therefore investigated the composition of the gut microbiome in the small and large intestine of these mice. The gut of the wt animals showed a greater diversity of bacterial species (Supplementary material 8: Supplementary Figure S2A) and a higher abundance of probiotic strains such as *Lactobacillus*, *Bifidobacterium* [57] and *Dubosiella* [58]. In contrast, the *Tcf7l2* cKO mice harbored a substantial concentration of bacterial strains, including *Enterococcus* and *Escherichia-Shigella*, which are commonly found in patients with inflammatory bowel disease (IBD) [59] (Fig. 1D and Supplementary material 8: Supplementary Figure S2B). In addition, the mice showed weight loss, indicating damage to the intestinal epithelium and the inability of the animals to absorb nutrients (Supplementary material 8: Supplementary Figure S2C). Strikingly, when the mice of the same genotype were treated with antibiotic vancomycin in the drinking water one week before Cre-mediated recombination of the floxed alleles, the epithelium, which previously had only a few foci of proliferating cells (Fig. 1AB), was completely restored and the mortality of the mice was significantly reduced (Fig. 1E). These mice also lost weight approximately 7–15 days after recombination, which is consistent with the effects of epithelial damage. However, their weights gradually returned to baseline, indicating the restoration of epithelial function (Supplementary material 8: Supplementary Figure S2C).

**Fig. 2:**
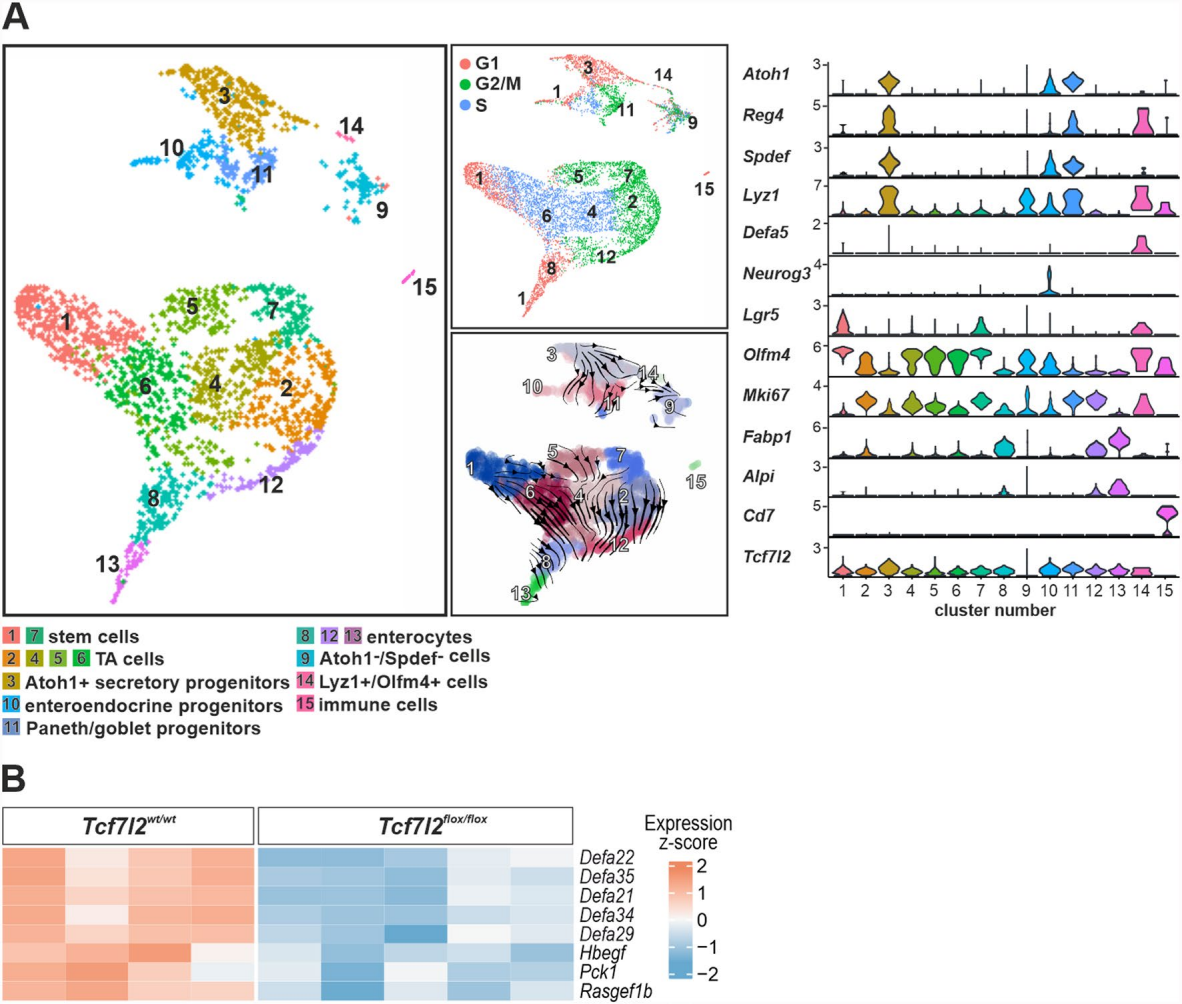
Transcriptome profiling of proliferating cells reveals a drop of α-defensin levels in *Tcf7l2*-deficient intestinal epithelium. **A** Single-cell transcriptome analysis of intestinal epithelial cells in *Tcf7l2*^*wt/wt*^*/Mki67*^*RFP/RFP*^*/Villin-CreER*^*T2*^ (*Tcf7l2*^*wt/wt*^) mice. Proliferating (i.e., Mki67-RFP-positive) epithelial cells were isolated and analyzed. The diagram on the left shows an UMAP (Uniform Manifold Approximation and Projection) visualization in which the cells are color-coded according to their identified cell type. For the full list of genes expressed in each cluster, see Additional file 2: Supplementary Table S2. The UMAP diagram on the top right side is colored based on the cell cycle phase. The right bottom UMAP diagram is an overlay of the UMAP clusters with arrows representing the lineage relationships between the cell types (RNA velocity). The violin diagram on the right shows the differential expression of key lineage markers in the identified clusters; the level of gene expression is indicated on the y-axis. Alpi, alkaline phosphatase, intestinal; Atoh1, atonal bHLH transcription factor 1; Defa5, defensin alpha 5; Fabp1, fatty acid binding protein 1; Lgr5, leucine-rich repeat-containing G protein-coupled receptor 5; Mki67, proliferation marker Ki-67; Neurog3, neurogenin 3; Olfm4, olfactomedin 4; Reg4, regenerating family member 4; Spdef, SAM pointed domain containing ETS transcription factor. **B** The bulk RNA sequencing heatmap shows differentially expressed antimicrobial/anti-inflammatory genes with color scaling indicating the expression level in *Tcf7l2*^*wt/wt*^ mice compared to the *Tcf7l2*^*flox/flox*^ tissue. Jejunum of the mice was analyzed 7 days after tamoxifen administration. Significant genes (adjusted p-value < 0.05 and |fold change (FC)|≥ 2) are shown. A complete list of differentially expressed genes can be found in Additional file 3: Supplementary Table S3. Hbegf, heparin binding EGF like growth factor; Pck1, phosphoenolpyruvate carboxykinase 1; Rasgef1b, RasGEF domain family member 1b

We then analyzed the phenotypes resulting after crossing the cKO *Tcf7l2* allele with the *Villin-CreER*^*T2*^ driver by transcriptional profiling. We examined the cellular expression program of the regenerating parts of the intestine, i.e. the hyperplastic crypts, compared to the proliferating cells of the crypts with intact *Tcf7l2* gene. Therefore, we crossed *Tcf7l2*^*flox/flox*^*/Villin-CreER*^*T2*^ mice in the homozygous state with the *Mki67*^*RFP*^ strain, which produces the red fluorescent protein TagRFP in frame at the C-terminus of Ki67. First, we performed single-cell (sc) RNA sequencing (RNA-seq) analysis of dividing, i.e., Mki67-RFP-positive, epithelial cells isolated from the middle part of the *Tcf7l2*^*wt/wt*^ small intestine. Since we intentionally did not correct for the cell cycle, some cell populations "secondarily" split into several clusters depending on the stage of the cell cycle (Fig. 2A; heatmap visualization is shown in Supplementary material 8: Supplementary Fig. S3A). The analysis also had to consider that the TagRFP protein is relatively stable and that some TagRFP-positive cells may no longer be active in the cell cycle. In any case, we observed three clusters of cells that are positive for the stem cell marker leucine-rich repeat-containing G protein-coupled receptor 5 (*Lgr5*) [60] and olfactomedin 4 (*Olfm4*) [61]. The first two (1 and 7) represent "classical" crypt base columnar (CBC) stem cells in phases G1/S and G2/M, respectively. The third cluster, number 14 (Lyz1^+^/Olfm4^+^ cells in Fig. 2A) is reminiscent of label-retaining cells (LRCs) that co-express stem cells [*Olfm4*, achaete-scute family bHLH transcription factor 2 (*Ascl2*)] and Paneth cell markers [defensins, *Reg4*, lysozyme 1 (*Lyz1*), mucosal pentraxin 2 (*Mptx2*), matrix metallopeptidase 7 (*Mmp7*)] [62]. Four clusters (2, 4, 5 and 6) represented transit amplifying (TA) cells; the cells in these and stem cell clusters had the highest levels of cell proliferation nuclear antigen (*PCNA*) and/or *Mki67*. Three clusters, 12, 8 and 13, represented the pathway to absorptive enterocytes [intestinal alkaline phosphatase (*Alpi*), sucrase-isomaltase (*Sis*), angiotensin-converting enzyme 2 (*Ace2*) and fatty acid binding protein 1 (*Fabp1*) expression] [63,64]. Three clusters [3,10,11] expressed the regulator of secretory cell fate, atonal bHLH transcription factor 1 (*Atoh1*) [65], serine peptidase inhibitor, Kazal type 4 (*Spink4*) and the Atoh1 target gene, SAM pointed domain containing ETS transcription factor (*Spdef*) [66]. Prediction of future gene expression of cells in clusters based on the ratio of spliced and unspliced mRNA (scRNA velocity) showed a transition from cells in cluster 3, which may represent the earliest secretory progenitor cells, to clusters 10 and 11, which represent slightly more developed cells of the enteroendocrine [positive for neurogenin 3 (*Neurog3*) [67] and glial cell line derived neurotrophic factor family receptor alpha 3 (*Gfra3*)] [68] and goblet/Paneth cells progenitors [markers anterior gradient 2 (*Agr2*), trefoil factor 3 (*Tff3*), Fc-γ binding protein (*Fcgbp*), *Muc2* for goblet and *Lyz1*, *Defa17/24*, *Mmp7* for Paneth cells] [63,69], respectively. The cells in cluster 9 were *Olfm4*- and *Lgr4*-positive and produced genes related to Paneth and goblet cells. However, the expression profile of these apparently secretory progenitor cells was very heterogeneous and some of these cells expressed enterocytic markers (Supplementary material 8: Supplementary Fig. S3A; Supplementary material 2: Supplementary Table S2). The majority of these cells were positive for *Spink4*, but did not produce the other typical secretory cell markers *Atoh1* and *Spdef*, which is why we named this cluster Atoh1^−^/Spdef^−^ cells. Interestingly, scRNA velocity analysis revealed the origin of the cells in cluster 14. Another notable observation was that the production of Tcf4 transcription factor by the cells in clusters 9 and 14 was essentially undetectable (Fig. 2A). This could indicate that these cells are Tcf4 independent. The cells in cluster 15 mainly produced immune cell markers. These cells (according to the expression profiles of CD3/CD8^+^ T cells; Supplementary material 2: Supplementary Table S2) were possibly (co-)isolated with intestinal cells.

We then performed bulk RNA-seq of Mki67-RFP-positive cells sorted from the *Tcf7l2* cKO mice, i.e., hyperproliferative epithelial cells in which the *Tcf7l2* gene remained intact. The RNA expression profile of cells isolated from mice without *CreER*^*T2*^ served as a control. The resulting bulk RNA-seq analysis showed that the dividing cells derived from the hyperproliferative parts of the epithelium had a similar expression profile to the dividing cells localized in normal crypts. This comparison revealed (only) 18 significantly increased genes encoding proteins (Supplementary material 3: Supplementary Table S3) included in the GSEA sets associated with cell growth and division or metabolic activities (Supplementary material 8: Supplementary Fig. S3B). Among the 12 downregulated genes, there were a total of five genes encoding various α-defensins and three genes encoding peptides involved in the antimicrobial or anti-inflammatory response, namely heparin-binding EGF-like growth factor (*Hbegf*), phosphoenolpyruvate carboxykinase 1 (*Pck1*) and RasGEF domain family member 1b (*Rasgef1b*) [70–72] (Fig. 2B). Reduced multiple *Defa* gene expression, particularly in the ileum, was also detected by quantitative RT-PCR (Supplementary material 8: Supplementary Fig. S3C). The above results show that the remaining crypts can restore the small intestinal epithelium when pathogenic intestinal bacterial strains are suppressed. The main reason for the failure of this restoration is a reduction in the expression of genes associated with the antimicrobial or anti-inflammatory functions of the epithelium.

### *Tcf7l2* gene controls the secretory cell type switching between Paneth and goblet cells in Defa6^+^ cells of the small intestine

**Fig. 3:**
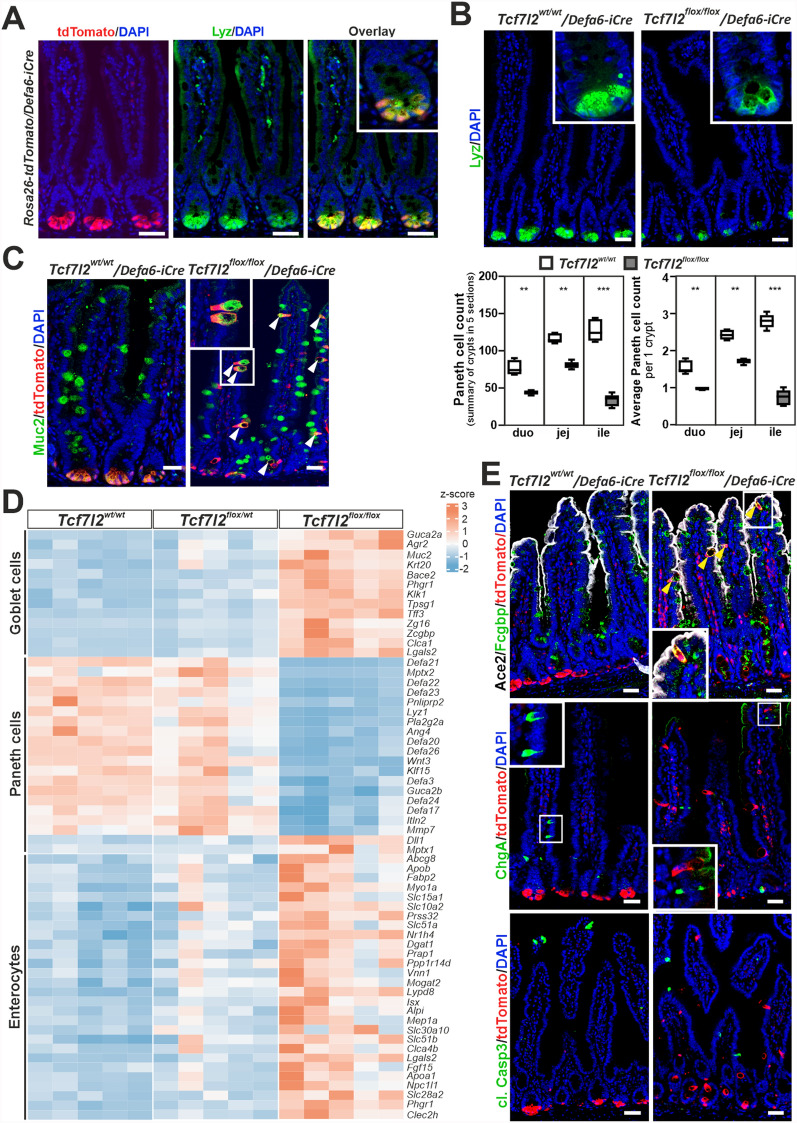
Comparative analysis of the Paneth cell morphology and distribution in *Tcf7l2* wt and knockout mice. Mouse lines *Tcf7l2*^*wt/wt*^*/Defa6-iCre* and *Tcf7l2*^*flox/flox*^*/Defa6-iCre* were used to analyze Defa6-tdTom cells after crossing with *ROSA26-tdTomato* reporter mice. **A** Confocal microscopy images show the co-localization of tdTomato (red signal) with lysozyme (Lyz; green signal) in intestinal sections. The overlay highlights the extent of co-localization. Scale bar: 50 µm. Magnified images are shown in the insets. **B** The upper panel shows Lyz expression in Paneth cells. The insets show the detailed cellular morphology and highlight the differences between Tcf4 wt (*Tcf7l2*^*wt/wt*^) and knockout (*Tcf7l2*^*flox/flox*^) conditions. The lower panels show quantitative analyses of the number of Paneth cells (based on Lyz positivity) in the duodenum (duo), jejunum (jej) and ileum (ile); n = 10. Statistical significance was determined using a one-way ANOVA test; **p < 0.01; ***p < 0.001. **C** Fluorescent micrographs showing mislocalized Muc2-positive *Tcf7l2*^*flox/flox*^ Defa6-tdTom cells (indicated by white arrowheads). Scale bar: 50 µm. **D** Heatmap of differentially expressed genes obtained by bulk RNA-seq from Defa6-tdTom cells with different genetic contexts (as indicated). Each row represents a gene and each column represents cells isolated from a single animal. Color intensity (blue to red) indicates the Z-score of gene expression levels and highlights differences and patterns of up- or downregulation. A complete list of differentially expressed genes can be found in Additional file 4: Supplementary Table S4. **E** Top: presence of the goblet marker Fc gamma binding protein (Fcgbp) in all *Tcf7l2*^*flox/flox*^ Defa6-tdTom cells on the villi (yellow arrowheads). The staining for the enterocyte marker angiotensin converting enzyme 2 (Ace2) is also shown (white signal). Center: lack of chromogranin A (ChgA) staining in Defa6-tdTom cells in wt or Tcf4-deficient intestine. Bottom: staining of cleaved caspase 3 (cl. Casp3) indicates apoptotic activity at the apical tips of small intestinal villi. Scale bar: 50 µm

In our model of intestinal crypt hyperplasia after partial loss of Tcf4, we observed misplaced cells that were positive for the Paneth cell marker lysozyme (Fig. 1C). This finding underlines the importance of the Wnt signaling pathway not only for homeostatic self-renewal but also for Paneth cell maturation. Thus, we investigated the effects of *Tcf7l2* gene inactivation using the *Defa6-iCre* mouse strain developed to target Paneth cells in the intestine [27]. In *Rosa26-tdTomato*/*Defa6-iCre* mice, we observed co-localization of tdTomato-labeled (Defa6-tdTom) cells with a Paneth cell marker lysozyme at the base of small intestinal crypts (Fig. 3A). Homozygous knockout of *Tcf7l2* in Defa6^+^ cells resulted in the absence of lysozyme granules and morphological changes indicative of vacuolization. In addition, the number of lysozyme-positive cells decreased significantly throughout the small intestine, especially in the ileum (Fig. 3B), correlating with the predominant loss of *Defa* expression in this intestinal segment. Although the overall architecture of the intestinal epithelium remained intact, a slight increase in proliferative activity was observed in the crypts. In addition, the area labeled with Olfm4, which denotes the stem cell compartment, increased in 2–3 months old mice, while it decreased in 1-year old mice (Supplementary material 8: Supplementary Fig. S4A), indicating a possible decrease in stem cell reserves due to the "efforts" to compensate for the loss of Paneth cells and maintain epithelial homeostasis [73]. Tracking of Defa6^+^ cells by tdTomato fluorescence showed that inactivation of *Tcf7l2* resulted in migration of Defa6-tdTom cells from the crypts into the villi, similar to the migration observed in other differentiated intestinal epithelial cells. These cells did not exhibit morphological characteristics of Paneth cells; in addition, they produced the goblet cell marker Muc2 (see Fig. 3C). The first signs of this cell migration were observed in the postnatal intestine as early as 15 days after birth (Supplementary material 8: Supplementary Fig. S4B).

Cells located in the lower part of the small intestinal crypts, including Paneth cells, produce surface marker CD24 [74]. Interestingly, FACS analysis showed virtually no CD24-negative Defa6-tdTom cells in wt mice (genotype: *Tcf7l2*^*wt/wt*^*/Rosa26-tdTomato/Defa6-iCre*). In contrast, CD24-negative Defa6-tdTom cells were observed in *Tcf7l2*^*flox/flox*^*/Rosa26-tdTomato/Defa6-iCre* mice (Supplementary material 8: Supplementary Fig. S5A). Isolation and genomic DNA analysis of both CD24-positive and CD24-negative tdTomato^+^ populations confirmed the presence of the recombinant *Tcf7l2*^*del(Ex5)*^ allele in *Tcf7l2* cKO mice, with increased presence in CD24-negative cells, indicating ongoing recombination and a decrease in functional Tcf4 protein in maturing Paneth cells (Supplementary material 8: Supplementary Fig. S5B). Quantitative RT-PCR analysis showed that the expression of *Tcf/Lef* genes was highest in wt mice in CD24-positive Defa6-tdTom cells, presumably Paneth cells. We detected a gradual decrease in *Tcf7l2* mRNA in both CD24-positive and CD24-negative Defa6-tdTom cell populations in *Tcf7l2* cKO mice. Interestingly, CD24^+^ Defa6-tdTom cells exhibited a significant decrease in expression of the *Tcf7* gene (encoding Tcf1), whereas expression of the paralogous *Tcf7l1* gene (encoding Tcf3) was increased. However, the decreased expression of the Wnt-responsive genes *Axin2* and naked cuticle homolog 1 (*Nkd1*) suggests that other members of the Tcf/Lef family are unable to maintain physiologic levels of Wnt signaling in the absence of functional Tcf4. Consequently, CD24-negative Defa6-tdTom cells also lost expression of crypt base-specific stem cell genes such as *Lgr5*, tumor necrosis factor receptor superfamily, member 19 (*Tnfrsf19*) [75], and *Olfm4* as well as the Paneth cell-specific genes *Defa24* and *Lyz1*. In contrast, we observed a strong increase in the expression of mRNAs characteristic of goblet cells, such as chloride channel accessory 1 (*Clca1*), *Muc2* and *Tff3* [76]. In addition, the expression of genes encoding enterocyte markers such as *Alpi*, *Fabp1* and *Sis* was increased [63,64] (Supplementary material 8: Supplementary Fig. S5C). To confirm these results, we performed bulk RNA-seq on Defa6-tdTom cells from combinations of *Tcf7l2* alleles, i.e., homozygous wt, heterozygous and homozygous cKO. While cells with heterozygous loss of *Tcf7l2* were almost identical in expression to wt cells, homozygous deletion of *Tcf7l2* resulted in marked changes in gene expression (Supplementary material 4: Supplementary Table S4). According to the Panglao database of cell type-specific gene expression [63], this was characterized by a marked loss of genes specific to Paneth cells and upregulation of genes characteristic of goblet cells and enterocytes (Fig. 3D). Histological staining revealed goblet marker Fcgbp positivity in all Defa6-tdTom cells at the villi in *Tcf7l2*^*flox/flox*^*/Rosa26-tdTomato/Defa6-iCre* mice. Given the distribution of the enterocyte marker Ace2 in most cells of the villi, it was difficult to determine whether the tdTom cells also co-express the Ace2 protein in addition to the goblet cell marker Fcgbp (Fig. 3E). In contrast, staining for chromogranin A (ChgA), which is specific for enteroendocrine cells, never overlapped with tdTomato labeling. In addition, visualization of cleaved caspase 3 showed that apoptosis in Defa6-tdTom cells occurs mainly at the villus tips regardless of Tcf4 status, indicating normal homeostatic cell renewal (Fig. 3E).

**Fig. 4:**
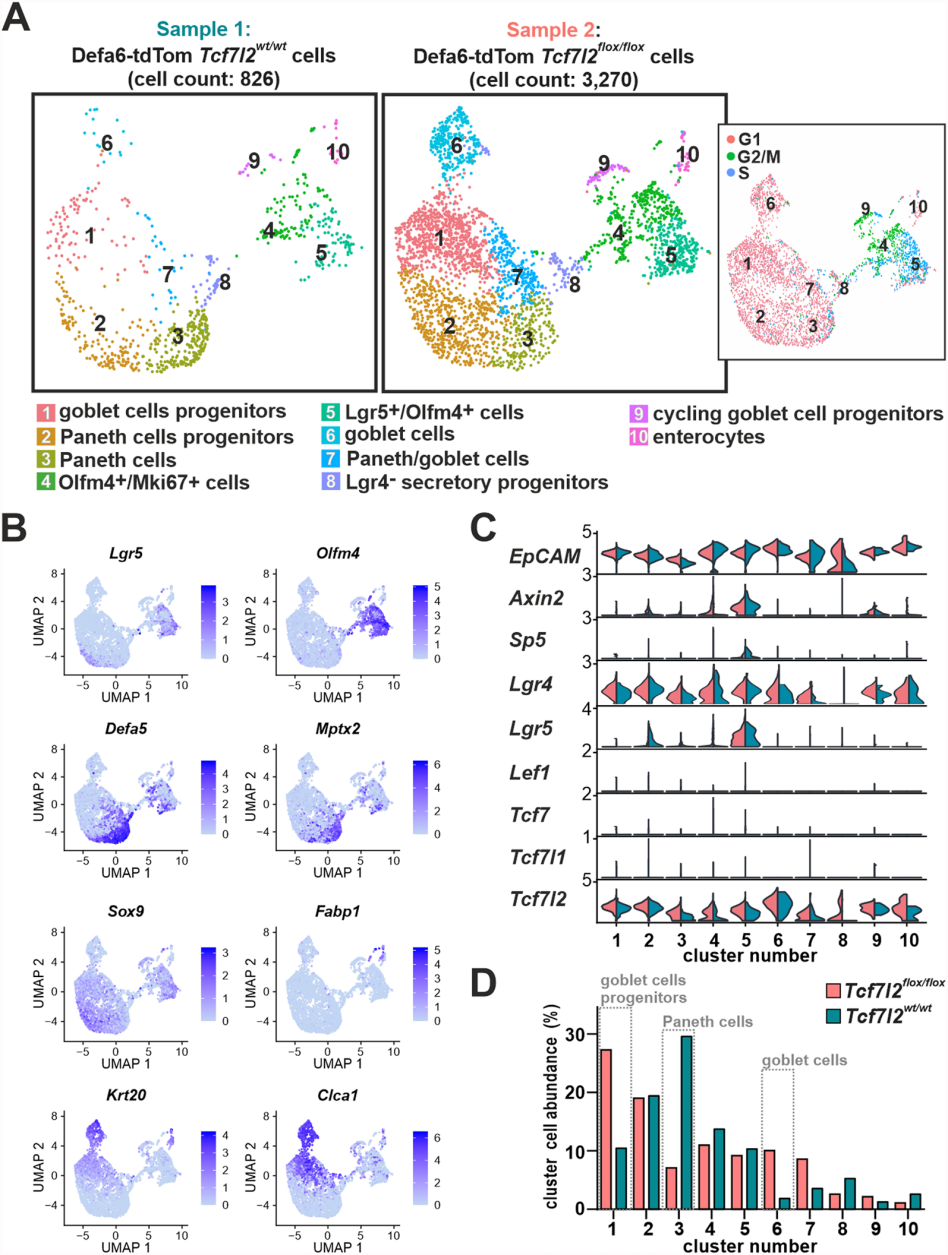
Tcf4-deficient cells leave the Paneth cell lineage and acquire gene expression characteristics of goblet cells. Single-cell RNA-seq data of Defa6-tdTom cells from the small intestine of different *Tcf7l2* genotypes. **A** Left, UMAP visualization of scRNA-seq clustering of Defa6-tdTom cells from the small intestine (jejunum) of mice with the wt (*Tcf7l2*^*wt/wt*^, sample 1) or knockout *Tcf7l2* (*Tcf7l2*^*flox/flox*^, sample 2) cells. Cell cycle analysis of Defa6-tdTom cells combined from both samples is shown in the inset. The cell clusters are numbered according to the amount of cells in the merged dataset. Cell types were assigned based on the genes specifically expressed in each cluster (see Additional file 5: Supplementary Table S5 for a complete list of genes). **B** UMAP feature plots showing the expression patterns of the different marker genes. The plots were derived from the merged dataset. Additional cell type markers are shown in Additional file 8: Supplementary Figure S5. Ace2, angiotensin-converting enzyme 2; Clca1, chloride channel accessory 1; Krt20, cytokeratin 20; Mptx2, mucosal pentraxin 2; Sox9, sex-determining region Y (SRY)-box 9. **C** Violin plots showing the expression level of epithelial cell adhesion molecule (*EpCAM*) gene as well as genes of the Tcf/Lef family in both Defa6-tdTom scRNA-seq samples. The expression levels of *Axin2* and *Sp5*, which indicate the status of Wnt signaling, are also shown in the identified cell clusters from panel A. **D** Frequency of cell types in the clusters of the two scRNA-seq samples. The percentages are plotted so that the total number of cells in a respective sample is 100%. Three cell types most changed between *Tcf7l2* wt and knockout samples are indicated by the gray dotted boxes. Lef1, lymphoid enhancer-binding factor 1

To comprehensively characterize the development of the Paneth cell lineage in the intestine and to show to what extent the loss of Tcf4 leads to its alteration, we performed scRNA-seq on Defa6-tdTom cells from the small intestine of *Tcf7l2*^*wt/wt*^*/Rosa26-tdTomato/Defa6-iCre* (Tcf4 wt; sample 1 in Fig. 4A) or *Tcf7l2*^*flox/flox*^*/Rosa26-tdTomato/Defa6-iCre* (Tcf4 cKO; sample 2 in Fig. 4A) mice. The combined analysis of both obtained datasets showed that Lgr5/Olfm4-positive proliferating (PCNA- and Mki67-positive) cells or rapidly dividing cells (producing large amounts of ribosomal proteins) [77,78] were present in three clusters: 4, 5 and 9 (Fig. 4ABC and Supplementary material 5: Supplementary Table S5). The cells in these clusters expressed target genes of the canonical Wnt signaling pathway *Axin2* and *Sp5* [79], indicating active Wnt signaling (Fig. 4C). Since *Mmp7* (and *Reg4*) was produced at significant levels in almost all cells in the scRNA-seq analysis, we were hesitant to name these cells as stem/TA cells. It should be noted that the transcriptional regulator *Ascl2* of intestinal stem cells [80] was preferentially produced in the cells of clusters 4 and 5 (Supplementary material 8: Supplementary Fig. S6AB). The cells in the other seven clusters were predominantly in the G1 phase of the cell cycle. Paneth cell progenitors expressing *Atoh1* and transcription factor *Sox9*, which is critical for Paneth cell differentiation [81], and general Paneth cell marker genes such as *Mmp7* and *Reg4*, and defensins were included in cluster 2. The cluster with significantly reduced cell number in the Tcf4 cKO sample was 3 (Fig. 4D). This cell cluster represents mature Paneth cells expressing a number of defensins, *Lyz1* and *Mptx2*. In addition, three clusters (1, 6 and 7) represented the goblet cell lineage dominantly expressing markers for goblet cells as well as cytokeratin 19 (*Krt19*), whose expression arises in the crypt compartment of TA cells [82]. Cluster number 6 represented mature goblet cells that were positive for a number of goblet cell markers (*Clca1*, *Muc2*, *Fcgbp*, *Spdef*, *Tff3*) and also for molecules that indicate the position of the cell on the villi, in particular *Krt20*, zymogen granule protein 16 (*Zg16*), and indoleamine 2,3-dioxygenase 1 (*Ido1*) [63,83]. This cluster, together with clusters 1 (goblet cell progenitors) and 7 (cells harboring both Paneth and goblet cell markers), was enriched in the Tcf4 cKO sample (Fig. 4C). Cluster number 8 contained a rather heterogeneous group of Defa24- Lyz1-positive cells. Low production of *Lgr4*, a close paralog of *Lgr5*, was typical of the cells in this cluster, as was the low to absent expression of the *Tcf7l2* gene. Therefore, we named cluster 8 as Lgr4^−^ secretory progenitors (Fig. 4C). It should be noted that in our experience, deletion of exon 5 in the *Tcf7l2* gene has no effect on the total amount of *Tcf7l2* mRNA in knockout cells. Since *Lgr4* is required for both cell proliferation and Paneth cell specification [84], these cells may represent a subpopulation of cells involved in the Paneth/goblet cell fate determination. The least abundant cluster 10 included enterocytes expressing *Ace2*, *Alpi*, *Fabp1/2*, *Krt20*, and *Sis*. These cells were present in both samples regardless of the *Tcf7l2* status; therefore, we suspect that this is due either to a rare stochastic transition of some secretory progenitor cells to the enterocytic lineage or, more likely, to a technical contamination of the FACS-isolated Defa6-tdTom cells by cells with high autofluorescence. In addition, the distribution of differentially expressed genes (determined by bulk RNA-seq) in the individual cell clusters identified in the scRNA-seq analysis confirmed the reduced abundance of the Paneth cell lineage and the increased presence of the goblet cell lineage observed in the Tcf4 cKO compared to the Tcf4 wt sample (Supplementary material 8: Supplementary Fig. S6C). Among the most altered biological processes in gene ontology (GO), we found signaling pathways related to immune and antimicrobial responses. In addition, pathways related to the localization and targeting of proteins in the endoplasmic reticulum (ER) or endoplasmic reticulum-associated degradation (ERAD) [85] were also significantly affected, indicating the presence of actively secreting cells in the samples studied (Supplementary material 8: Supplementary Fig. S6D).

In summary, Defa6-iCre expression in the intestinal crypt begins in secretory progenitor cells. The function of Tcf4 is crucial for the specification of the Paneth cell lineage. Disruption of this function leads to the differentiation of secretory progenitor cells into goblet cells, which is associated with the loss of Paneth cells.

### Multiple source cells of the Wnt3 ligand were identified in the epithelium of the small intestine

**Fig. 5:**
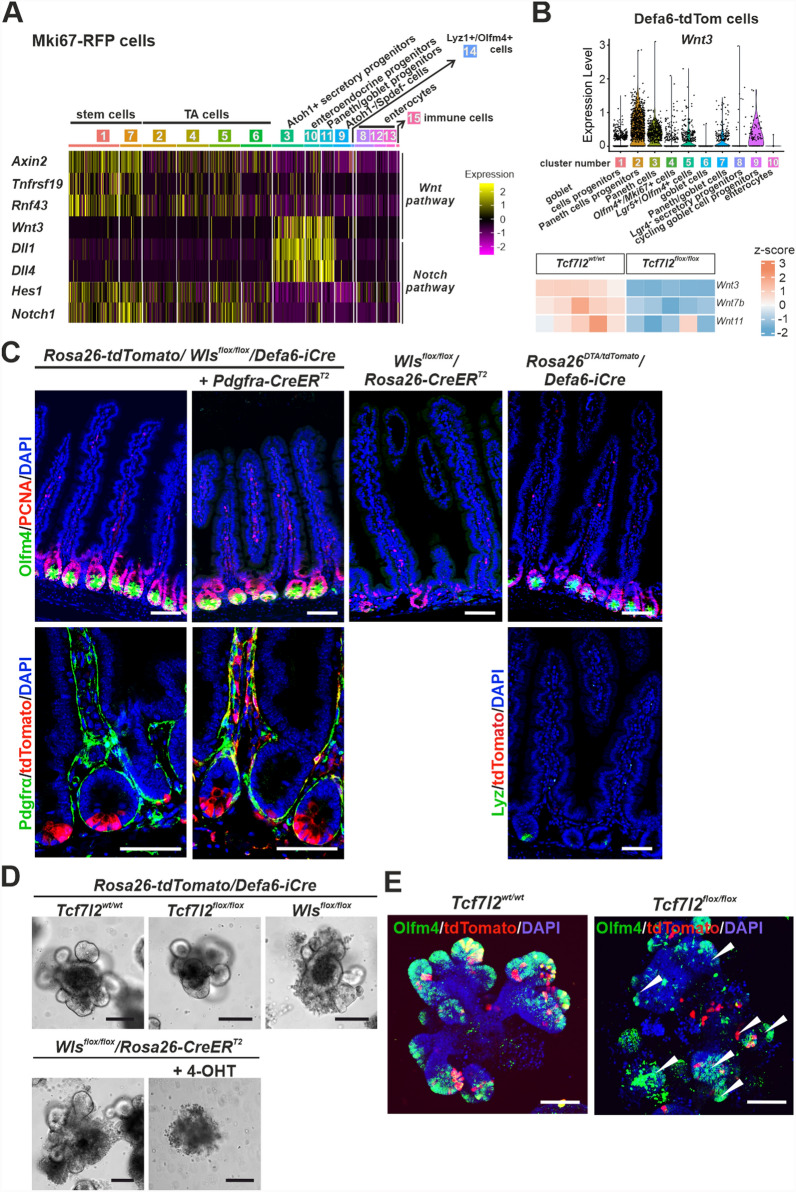
Different cell types that produce Wnt ligands in the small intestinal epithelium. **A** Heatmap with scaled expression of genes involved in the Wnt (top) and Notch (bottom) signaling pathways. Expression levels are color-coded, with yellow indicating high expression and purple indicating low expression. Dll1/4, Delta-like canonical Notch ligand 1/4; Hes1, Hes family basic helix-loop-helix (bHLH) transcription factor 1; Notch1, Notch homolog protein 1; Rnf43, ring finger protein 43; Tnfrsf19, tumor necrosis factor receptor superfamily, member 19. **B** Top, violin plot showing the expression levels of *Wnt3* in different cell clusters of Defa6-tdTom cells. Dots represent individual cells. Bottom, heatmap showing the expression of indicated Wnt ligands in Tcf4 wt Defa-tdTom cells. In cells with homozygous Tcf4 cKO, downregulation of Wnt ligands was detected. **C** Immunohistochemical analysis of the crypt compartment after genetic knockout of the *Wls* gene, 8 days after recombination, using indicated Cre recombinase-expressing mouse strains. Left and center, images showing the absence of Olfm4 and reduced PCNA staining observed only after inhibition of Wnt ligand secretion in all epithelial and subepithelial cells. Right, additional panel of Muc2 and tdTomato staining highlighting the mucus-producing goblet cells in the intestinal crypts and showing the effects of Paneth cell depletion using diphtheria toxin A (DTA). Bottom, documentation of recombination in Defa6^+^ and Pdgfrα^+^ cells using tdTomato reporter protein fluorescence. The scale bars correspond to 0.15 mm. Wls, Wntless. **D** Growth of intestinal organoids from crypts containing *Tcf7l2* wt, *Tcf7l2* cKO, or *Wls* cKO in Defa6-tdTom cells (upper panel) or *Wls* cKO in all cells induced by administration of 4-hydroxytamoxifen (4-OHT) in culture medium (bottom panel). **E** Representative fluorescence images of *Tcf7l2* wt and cKO organoids showing Olfm4-positive crypt compartments and Defa6-tdTom cells. The scale bars correspond to 0.1 mm

Paneth cells not only provide antimicrobial protection but also create a microenvironment known as a niche for ISCs and produce Wnt3 (Fig. 5A). An interesting, although somewhat expected, finding was that the secretory progenitor cells, regardless of the type of compartment, expressed the delta-like canonical Notch ligand (*Dll*) *1/4* (Fig. 5A). In contrast, cells in the stem cell compartment produced the receptor of this pathway, *Notch1*, and, according to the expression of the target gene of this pathway hes family bHLH transcription factor 1 (*Hes1*), actively signaled from the receptor [86]. A similar situation was observed for Wnt signaling. The *Wnt3* ligand was expressed in secretory progenitor cells, while the target genes of this pathway *Axin2*, *Tnfrsf19* and ring finger protein 43 (*Rnf43*) [87] were mainly produced in stem cells and to some extent also in the progenitor compartment (Fig. 5A). We also detected *Wnt3* expression in most Defa6-tdTom cell types, i.e., in both secretory progenitor cells and Paneth cells. In addition to *Wnt3*, we observed expression of the canonical ligand *Wnt7b* [88] and the non-canonical *Wnt11* [89, 90]. Bulk RNA-seq showed that inactivation of the T*cf7l2* gene in Defa6-tdTom cells resulted in decreased expression of these Wnts (Fig. 5B and Supplementary material 4: Supplementary Table S4).

The loss of Paneth cells after inactivation of the *Tcf7l2* gene had no effect on epithelial renewal, which was expected since it had already been predicted and subsequently shown that Paneth cells are not the only source of Wnt ligands in the intestine [11,13,91]. To achieve complete loss of Wnt ligand secretion in Defa6-tdTom cells, we used a mouse strain that allows conditional inactivation of the Wntless (*Wls*) gene, which encodes a transmembrane protein essential for Wnt ligand secretion from the cell [92]. Similar to the significant reduction in Wnt ligand expression in mice of the *Tcf7l2*^*flox/flox*^*/Defa6-iCre* genotype, the loss of Wnt ligands secretion in *Wls*^*flox/flox*^*/Defa6-iCre* mice had no effect on stem cell numbers and epithelial homeostatic renewal. Likewise, no morphological changes of the epithelium were observed after depletion of Paneth cells in *Rosa26*^*DTA/tdTomato*^*/Defa6-iCre* mice caused by production of diphtheria toxin A (DTA), a so-called suicide gene, from the *Rosa26* locus [25] (Fig. 5C). Recently, subepithelial mesenchymal cells have been shown to serve as a secondary source of Wnt signaling for ISCs [13,14]. Therefore, we subsequently inhibited Wnt ligand secretion in mesenchymal cells using mice that produce CreER^T2^ recombinase in mesenchymal cells producing platelet-derived growth factor receptor alpha (*Pdgfrα*) [93,94]. Surprisingly, the simultaneous blocking of Wnt ligand secretion in Defa6^+^ and Pdgfrα^+^ cells did not lead to a loss of intestinal stem and proliferating cells. In contrast, inactivation of the *Wls* gene with the *Rosa26-CreER*^*T2*^ driver, which enables deletion of the floxed allele in all cell types, led to a significant reduction in the number of proliferating cells in the crypts of the small intestine (Fig. 5C).

We then established organoid cultures and compared the effects of inactivating the *Tcf7l2* gene in Defa6-tdTom cells, either with the *Defa6-iCre* transgene or with inactivation of the *Wls* gene with the *Defa6-iCre* or *Rosa26-CreER*^*T2*^ driver. While organoids with inhibited Wnt secretion in all cells (*Wls*^*flox/flox*^*/Rosa26-CreER*^*T2*^; Fig. 5D, bottom) collapsed after the first passage, organoids with *Tcf7l2* or *Wls* inactivation specifically in Defa6⁺ cells continued to grow and displayed a branched morphology, resembling wt organoids, for at least five passages. However, *Wls* cKO organoids had dead cells in their vicinity (Fig. 5D, top). However, the "crypts" of organoids with the *Tcf7l2* cKO allele were partially disordered and had fewer Olfm4-positive cells (Fig. 5E, white arrowheads). In addition, tdTomato-positive cells were more centrally located, i.e., in the organoid region that contained more derived cell types (Fig. 5E). Thus, this phenotype resembled the situation observed in in vivo experiments.

In summary, Paneth cells and precursors of the secretory lineage are the main source of Wnt ligands in the small intestine, which is consistent with published results. However, the loss of these cells associated with blocking Wnt ligand secretion in mesenchymal cells is not consistent with the phenotype observed when the Wnt signaling pathway was inhibited in the intestinal crypts.

### The gene expression program regulating antimicrobial peptide production is activated in early intestinal adenomas

**Fig. 6:**
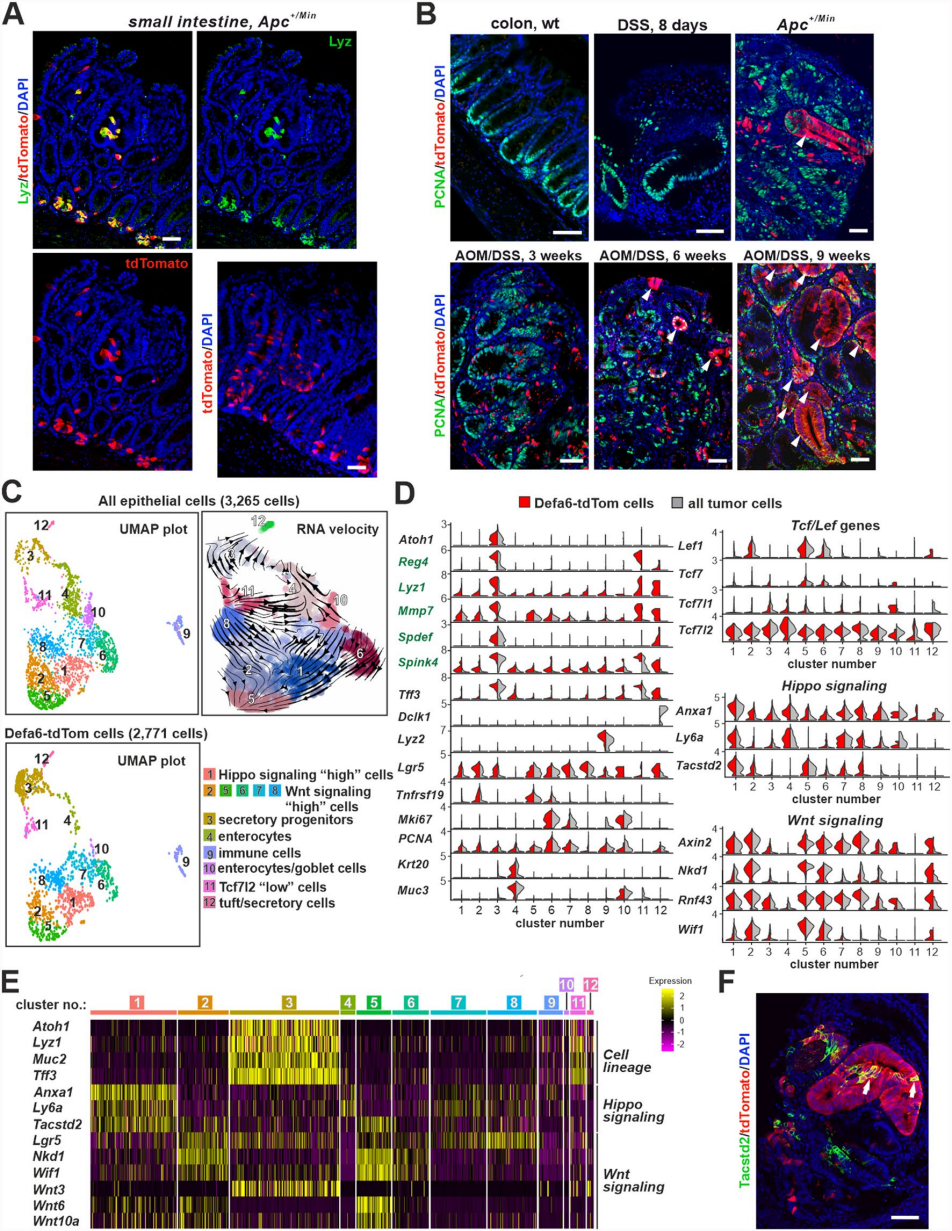
Activation of α-defensin expression during the development of the small intestinal and colonic adenomas. **A** Presence of Defa6-tdTom⁺ cells (red signal) in small intestinal adenomas of *Apc*^+*/Min*^ mice. These cells are mislocalized from the crypts but mostly retain lysozyme positivity (green signal). Scale bar: 100 µm." **B** The appearance of Defa6-tdTom cells is associated with tumorigenesis in the colon. Representative fluorescence microscopy images of cells stained with an antibody against PCNA (green signal) and tdTomato (red signal). Note that the red fluorescent signal was observed either in mice treated with the mutagen azozymethane (AOM) in combination with colitis-inducing sodium dextran sulfate (DSS) or in the colon tumor developed in *Apc*^+*/Min*^ mice. In contrast, "red" cells are present neither in the healthy colonic epithelium (wt) nor in the epithelium 8 days after the DSS treatment, i.e., without mutagen. The red glandular structures are formed 6 and 9 weeks after AOM/DSS treatment and in tumors of the *Apc*^+*/Min*^ mice (white arrowheads). Scale bar: 150 µm. **C** Single-cell RNA-seq analysis of epithelial cells from dissected colon tumors in *Apc*^+*/Min*^ mice. Cell clusters are numbered according to the cell abundance; UMAP visualizations show all epithelial tumor cells (top) and Defa6-tdTom cells (bottom). The top right diagram is an overlay of the UMAP clusters with arrows representing the lineage relationships between the cell types (RNA velocity). For better visibility, the diagram has been expanded spatially and cluster 9 (immune cells) is not shown. A complete list of cluster marker genes can be found in Additional file 6: Supplementary Table S6. **D** Left, the expression of marker genes in different cell clusters is shown in the violin plots, with the level of gene expression indicated on the y-axis. Genes with increased expression in Defa6-tdTom tumor cells are shown in green. Right, analysis of the Wnt signaling pathway components in individual cell clusters. Each violin plot represents the mean expression level for the Wnt signaling target genes, the genes encoding the Wnt signaling nuclear mediators Tcf/Lef or genes activated upon the Hippo pathway inhibition. Expression in all epithelial tumor cells is shown in gray, Defa6-tdTom tumor cells are shown in red. **E** Heatmap showing scaled expression of indicated genes in sorted Defa6-tdTom tumor cells; cluster numbers were obtained from the combined analysis of total tumor cells and Defa6-iCre-labeled cells. Expression levels are color-coded, with yellow indicating high expression. **F** Representative microscopy images of cells stained with an antibody against Tacst2 and tdTomato. Note the Tacstd2-positive cells in the red glandular structure (white arrows). Scale bar: 150 µm

In the small intestine, we observed that the absence of Tcf4 led to reduced expression of antimicrobial peptides in Defa6⁺ cells. This finding prompted us to investigate whether, conversely, the expression of antimicrobial peptides in tumors could be upregulated by abnormally enhanced Wnt signaling. To test this, we examined small intestinal adenomas in *Apc*^+*/Min*^ mice crossed with *Rosa26-tdTomato/Defa6-iCre* mice and detected scattered Defa6-tdTom⁺ cells within the adenomas (Fig. 6A). Some of these cells were positive for lysozyme (Fig. 6A, top images), making it unclear whether they were dislocated Paneth cells or cells that had arisen within the adenoma.

To clarify this, we focused on adenomas in the colon. In healthy adult *Rosa26-tdTomato/Defa6-iCre* mice, red fluorescence was not detected in the normal colon epithelium (Fig. 6B, first image) or in the epithelium 8 days after colitis induction by DSS treatment (Fig. 6B, second image). However, we observed the presence of Defa6-tdTom⁺ cells in mice when DSS administration was preceded by treatment with the mutagen azoxymethane (AOM), as well as in colon adenomas in *Apc*^+*/Min*^ mice (Fig. 6B). Similar to small adenomas in the small intestine of *Apc*^+*/Min*^ mice, adenomas examined three weeks after AOM/DSS treatment contained only scattered Defa6-tdTom⁺ cells. In contrast, the more advanced colon adenomas of *Apc*^+*/Min*^ mice showed clusters or glands of Defa6-tdTom cells in addition to scattered cells. In addition, six weeks after AOM/DSS treatment, glandular structures consisting of Defa6-tdTom cells began to form (Fig. 6B).

We used FACS to isolate either all epithelial cell adhesion molecule (EpCAM)^+^ or Defa6-tdTom/EpCAM^+^ cells from dissected colon tumors; the sorting strategy is shown in Supplementary material 8: Supplementary Fig. S7A. We then analyzed these cells by scRNA-seq. The obtained datasets were merged and clusters were identified based on the previously described cell type-specific gene expression profiles [95–97] (Fig. 6CD). A complete list of genes expressed in the individual clusters can be found in Supplementary material 6: Supplementary Table S6.

As expected, many of the profiled cells were proliferating, as shown by the expression of *PCNA* and/or *Mki67*, albeit to varying degrees (Fig. 6D and Supplementary material 6: Supplementary Table S6). Almost all cell clusters expressed the transcription factor *Sox9*, which has previously been associated with tumorigenesis in the colon epithelium [98,99]. Another gene that was expressed almost in all clusters was *Lgr5*. In several clusters (nos. 2, 5 and 6), we observed co-expression of *Lgr5* and another intestinal stem cell marker, *Tnfrsf19*. As molecular basis of tumorigenesis in *Apc*^+*/Min*^ mice is the abnormal activation of the canonical Wnt signaling pathway mediated by β-catenin (reviewed in [100]), we expected tumor cells to express Wnt signaling target genes. This assumption was confirmed by the observation that the majority of cells in the clusters examined expressed the Wnt signaling target gene *Axin2*, *Rnf43*, *Sp5*, Wnt inhibitory factor 1 (*Wif1*), and zinc and ring finger 3 (*Znrf3*), which had previously been identified as a target gene in intestinal tumors [101] (Fig. 6DE). In clusters 2, 5–8, the relatively highest amounts of the canonical target gene *Axin2* and of the *Rnf43* gene were produced. Apart from the overproduction of *Lgr5*, these clusters did not show differential expression of the so-called cell line-specific genes, which is why we designated these clusters as Wnt signaling "high" cells. Interestingly, RNA velocity analysis revealed a cellular trajectory of cells in the above clusters toward cluster 5, and cells in this cluster showed the highest expression of many target genes of the Wnt signaling pathway, including *Lef1*, which is also activated by the Wnt signaling pathway in intestinal epithelial cells [102]. On the way to cluster 5, there were also cells from cluster 1, which was characterized by increased expression of genes regulated by the Hippo signaling pathway. These included the annexin A1 gene (*Anxa1*) and genes coding for the so-called oncofetal (regenerative) stem cell antigen Sca1 (encoded by lymphocyte antigen-6; *Ly6a*) and tumor-associated calcium signal transducer 2 (*Tacstd2*) (Fig. 6DE) [103–105]. Interestingly, the cells positive for *Tacstd2* formed clusters in the tumor including glandular structures labeled with *Defa6-iCre*; however, the scattered Defa6-tdTom cells were predominantly Tacstd2-negative (Fig. 6F). Furthermore, our expression analyses of all four members of the Tcf/Lef family members confirmed previous findings [106] that Tcf4 is the predominant nuclear mediator of the Wnt signaling pathway in the colon (Fig. 6D). Cell clusters that were the most overrepresented in samples containing all epithelial tumor cells were clusters 4 and 10, with cells in cluster 4 expressing *Krt20*, *Muc3*, and enterocytes markers deleted in malignant brain tumors 1 (*Dmbt1*) and gasdermin C3 (*Gsdmc3*). GSEA showed that this cluster indeed contained enterocytes. This cluster, although less abundant, was also clearly defined in the Defa6-tdTom cells, and RNA velocity analysis showed it to be terminal. Cluster 10 appeared more heterogeneous and contained markers of enterocytes and goblet cells. Another heterogeneous cluster was cluster 11, which was characterized by the expression of secretory lineage marker *Spink4* and production of markers for Paneth cells (*Lyz1*, *Mmp7*) and goblet cells [*Agr2*, *Muc2*, seminal vesicle antigen-like 1 (*Sval1*) and *Tff3*]. The common feature of the cells in this cluster was the absence of transcripts for the *Atoh1* and *Spdef* genes and low expression of *Tcf7l2* and other members of the Tcf/Lef family (we named the cluster Tcf7l2 “low” cells). Tuft cell precursors expressing doublecortin-like kinase 1 (*Dclk1*) [107], together with tdTomato-positive secretory precursors, formed cluster 12. Immune cells, which were presumably isolated together with epithelial cells (positive for *Lyz2* and various other markers such as *CD14* and immunoglobulin kappa constant, *Igkc*), were clearly delineated as cluster 9. Cell cluster 3, which we labeled "secretory progenitor cells" based on the expression of *Atoh1* [66] was significantly enriched in Defa6-tdTom cells (29% vs. 9% in all epithelial cells isolated from the dissected tumors) (Supplementary material 8: Supplementary Fig. S7B). In addition to *Atoh1*, cells in this cluster also expressed *Lgr5*, *Sox9* and a number of goblet cell markers, such as the aforementioned *Tff3*, *Muc2* and *Agr2*, as well as *Spdef* [108] and *Spink4* [109]. The cells in this cluster also almost exclusively expressed *Wnt3* and genes typical of Paneth cells (e.g., *Lyz1*, *Mmp7*) or colonic enteroendocrine cells (*Reg4*) [110] (Supplementary material 6: Supplementary Table S6). It should be noted that the latter two genes are produced by DCS (see Introduction) and were enriched in Defa6-tdTom tumor cells (Fig. 6D; genes in green).

### Loss of Tcf4 in colon tumor cells promotes goblet cell differentiation

**Fig. 7:**
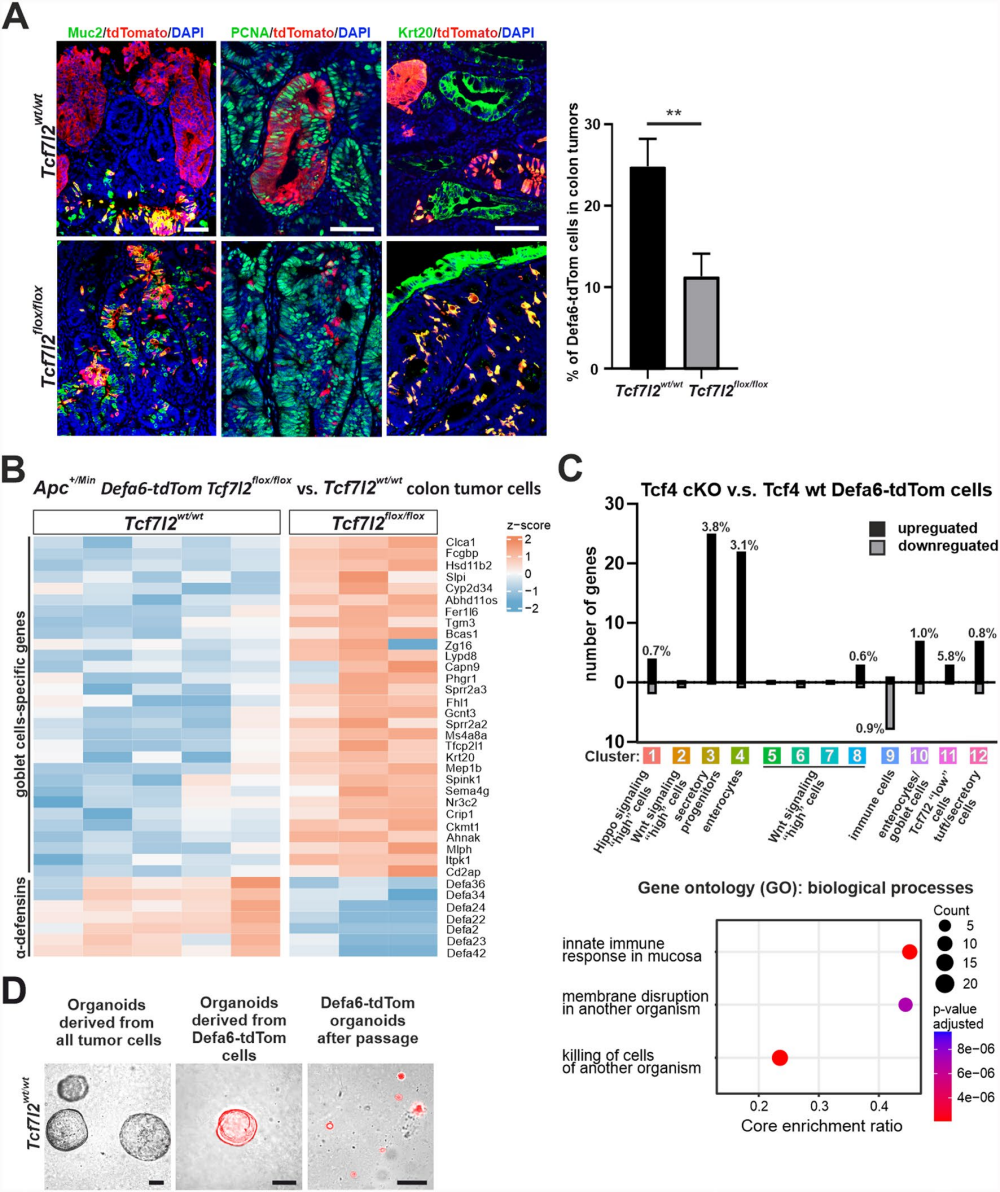
Changes in α-defensin expression and goblet cell phenotype due to disruption of the *Tcf7l2* gene. Mice with a *Tcf7l2*^*flox/flox*^ genotype were crossed with *Apc*^+*/Min*^*/ROSA26-tdTomato/Defa6-iCre* mice to generate progeny with Defa6-tdTom cells carrying either wt *Tcf7l2* alleles or a *Tcf7l2* cKO. **A** Left, fluorescence microscopy shows co-localization of Defa6-tdTom cells with antibodies against Muc2, PCNA and Krt20. Scale bar: 0.15 mm. Right, quantification of Defa6-tdTom cells in colon tumors. The red fluorescent signal was quantified in tumors harboring wt *Tcf7l2* (n = 16) or *Tcf7l2* cKO (n = 10). Data are represented as mean ± SEM. Statistical significance was determined using the one-way ANOVA test; **p < 0.01. **B** Heatmap showing scaled expression obtained by bulk RNA-seq of Defa6-tdTom cells isolated from colon adenomas comparing *Tcf7l2*^*flox/flox*^ to *Tcf7l2*^*wt/wt*^ colon tumor cells. Differential expression of genes associated with secretory goblet cells and Reg4^+^ cells is indicated. For a complete list of differentially expressed genes see Additional file 7: Supplementary Table S7. **C** Top, overlaps between differentially expressed genes in Defa6-tdTom tumor cells carrying *Tcf7l2* cKO (compared to cells with wt *Tcf7l2*) and genes significantly enriched in cellular clusters obtained after scRNA-seq analysis of Defa6-tdTom cells isolated from colorectal tumors. The values next to the bars indicate the percentage of differentially expressed genes in each cluster. Bottom, GO biological processes enriched in Tcf7l2-deficient Defa6-tdTom cells are depicted, showing the three most affected pathways based on adjusted p-values. **D** Representative images of organoid cultures derived from both non-fluorescent total tumor cells and Defa6-tdTom cells isolated from colon tumors of *Tcf7l2*^*wt/wt*^ mice. To promote tumorigenesis, mice were treated with AOM/DSS for 10 weeks prior to tumor harvesting. After sorting, cells were encapsulated in Matrigel and cultured in complete organoid culture medium (ENR) containing Wnt surrogate ligand. Images were taken after 7 days of initial culture or 4 days after the first passage. Scale bar: 0.1 mm

Next, we investigated the effects of Tcf4 deficiency in tumor cells expressing Defa6-iCre. We crossed *Tcf7l2*^*flox/flox*^ mice with *Apc*^+*/Min*^*/ROSA26-tdTomato/Defa6-iCre* mice and analyzed the tumors that developed in the offspring with the *Tcf7l2*^*flox/flox*^*/Apc*^+*/Min*^*/ROSA26-tdTomato/Defa6-iCre* genotype. Mice with the *Tcf7l2*^*wt/wt*^*/Apc*^+*/Min*^*/ROSA26-tdTomato/Defa6-iCre* genotype served as controls. In Tcf4-deficient adenomas, we observed a significant decrease in the number of tdTomato-positive glandular structures. The red fluorescent cells were scattered throughout the tumor tissue (Fig. 7A; Supplementary material 8: Supplementary Fig. S7C). Subsequent immunohistochemical analysis revealed that the Defa6-tdTom cells in Tcf4 knockout tumors had reduced proliferation, as evidenced by the absence of PCNA protein production. However, Krt20 and Muc2 were detected in these cells, indicating epithelial cell differentiation. It is important to note that strongly Krt20-positive cells were identified within the "red" cells in the glandular structures of wt Tcf4 mice. We also detected co-expression of Muc2 and tdTomato in wt Tcf4 mice, but mainly outside the glandular structures (Fig. 6CD).

We next sorted and analyzed Defa6-tdTom tumor cells from the colon tumors of *Apc*^+*/Min*^*/ROSA26-tdTomato/Defa6-iCre* and *Tcf7l2*^*flox/flox*^*/Apc*^+*/Min*^*/ROSA26-tdTomato/Defa6-iCre* mice using bulk RNA-seq. We found downregulation of several α-defensin genes and upregulation of genes predominantly associated with goblet cells [83] (see Fig. 7B for a heatmap representation and Supplementary material 7: Supplementary Table S7 for the full list of differentially expressed genes). When comparing the differentially expressed genes from the bulk RNA-seq with the cellular clusters derived from the scRNA-seq, it was found that the largest overlap among the genes with increased expression after inactivation of the *Tcf7l2* gene was found in clusters 3 and 4. The overlap of genes with decreased expression (after inactivation of *Tcf7l2*) was minimal, unless we used cluster number 9 (immune cells) (Fig. 7C, top). In addition, GSEA revealed that the most altered biological processes were related to "innate immune response in mucosa", "membrane disruption in another organism and antimicrobial response" and "killing of cells of another organism" (Fig. 7C, bottom). It is important to emphasize that these terms were identified after a marked decrease in α-defensin gene expression in Tcf4-deficient tumors. After FACS isolation and Matrigel culture of colon adenoma cells, we confirmed the ability of Defa6-tdTom cells to form organoids. Remarkably, the organoid-forming efficiency of Defa6-tdTom cells was significantly reduced compared to non-tdTomato-labeled tumor cells, with the former forming smaller and slower proliferating organoids after the first passage (Fig. 7D).

In summary, colorectal adenomas contain secretory lineage cells similar to Paneth cells. These cells produce antimicrobial peptides and the Wnt3 ligand. After the loss of Tcf4, these cells take on the characteristics of goblet cells and lose the expression of α-defensin, which can subsequently affect the characteristics of the tumor and its interaction with the colon microbiome.

## Discussion

Our study shows that Tcf4 plays a crucial role in regulating secretory cell fate and antimicrobial peptide production in both healthy intestinal epithelium and colorectal tumors. In the small intestine, the absence of Tcf4 led to the depletion of Paneth cells, a shift in secretory progenitor cell differentiation toward goblet cells, and alterations in the gut microbiota composition. Despite this loss, epithelial renewal was maintained due to alternative Wnt ligand sources. In colorectal tumors, we identified a subpopulation of secretory lineage cells that express antimicrobial peptides and Wnt3 and resemble Paneth cells. However, the loss of Tcf4 in these cells resulted in a phenotypic shift toward goblet cell-like differentiation, reduced proliferation, and impaired organoid formation. Transcriptomic analyses also revealed downregulation of α-defensins and upregulation of goblet cell-associated genes, with significant enrichment of signaling pathways related to immune and antimicrobial responses.

We used partial (incomplete) inactivation of the *Tcf12* gene to investigate the regenerative potential of the small intestinal crypt compartment. We observed ongoing epithelial recovery that was insufficient for animal survival (Fig. 1ABE). Treatment with vancomycin restored epithelial integrity and significantly reduced mortality in mice with (partial) Tcf4 inactivation. It is evident that this phenomenon is likely related to the rapid onset of dysbiosis, which we documented by sequential analysis of bacteria colonizing the digestive tract (Fig. 1D and Supplementary material 8: Supplementary Figure S2).

Another notable observation was the absence (low expression) of *Tcf/Lef* mRNA in some clusters of Mki67-RFP-positive cells. This particularly affected cluster 9 (Fig. 2A) containing early secretory progenitor cells. Interestingly, cluster 11 also showed very low expression of the *Tcf7l2* gene in cells isolated from colorectal tumors (Fig. 6D). In addition, the expression of other members of the Tcf/Lef family was almost undetectable. It is also interesting that we found the lowest activity of the Wnt signaling pathway in the mentioned cluster (No. 11), measured by the expression of the target genes of this pathway. This finding would imply that in addition to the production of Wnt ligands and other signaling mediators of this pathway [111–113], the level of canonical Wnt signaling in intestinal cells could be modulated by regulating the expression of *Tcf7l2*. This type of regulation, which to our knowledge has not been previously documented, may play a (significant) role, especially in cells at the bottom of the crypt that are likely exposed to Wnt ligands from neighboring cells (Fig. 5AB).

Our scRNA-seq analysis of Defa6-tdTom cells showed that continuous loss of Tcf4 in the adult intestine leads to an increase in goblet cell progenitors and a decrease in the Paneth cell lineage. Cells with a so-called intermediate phenotype (production of mucin and markers typical of Paneth cells), which were observed when using the *Villin-CreERT2* driver [8], were detected at a low quantity (Fig. 4; cluster 7). We were also able to detect them in the developing intestine of young mice (P15; Supplementary material 8: Supplementary Fig. S4B). Interestingly, immunohistochemical staining did not reveal positivity for lysozyme in any cells on the villi. Therefore, we conclude that inactivation of Tcf4 leads to cessation of Paneth cell development, and one of the ways to lose existing (mature) cells is their migration out of the crypt. However, bulk RNA-seq also revealed that Tcf4-deficient Defa6-tdTom cells produce markers for enterocytes. Does this indicate a different type of cell with a "mixed" phenotype? Probably not. Comparing the expression levels of individual marker genes in the dataset obtained, it becomes clear (Supplementary material 5: Supplementary Table S5) that genes typical of different cell lineages of intestinal epithelial cells are not only typical of a particular cell type but are also produced by other cell types, albeit at lower levels. Thus, it is not about the de novo expression of a specific marker, in this case an enterocyte marker, but about the differences in the expression of a specific gene when comparing distinct cell lines.

Recent research has identified a unique subpopulation of goblet cells in the intestinal epithelium, the so-called intercrypt goblet cells (icGCs). These cells are essential for maintaining the mucus barrier in the colon. In contrast to the conventional goblet cells, the icGCs are located on the colon surface between the crypts and secrete a specific type of mucus. A similar (sub)population of goblet cells has been found in the distal part of the small intestine [83]. A typical feature of these cells is their common expression of markers for goblet cells and enterocytes. This suggests that some Defa6-tdTom cells may be non-canonical goblet cells.

Another intriguing question is why only a small proportion of the goblet cells on the villi are positive for tdTomato. This can be explained by partial recombination of the reporter allele in the pool of progenitor cells that differentiate into goblet cells. Given the constitutive activity of Defa6-iCre and the high efficiency of recombination in Paneth cells, this seems unlikely. Rather, we are inclined to think that the *Defa6-Cre* driver is expressed in a restricted subset of progenitor cells that differentiate into (a subset of) goblet cells; these cells are not dependent on Tcf4 function.

In the small intestine, Wnt ligands play a pivotal role in regulating stem cell maintenance and directing cellular differentiation and proliferation. The production of these ligands primarily occurs in Paneth cells secreting Wnt3, a key ligand that supports the stem cell population and facilitates the stem cell-driven renewal of the intestinal epithelium [74]. Additionally, subepithelial myofibroblasts, located just outside the epithelial cell layer, also contribute to the Wnt ligand pool, particularly Wnt2/2b, thereby supporting the crypt stem cell environment from the underlying mesenchyme [14]. Our analysis has shown that Wnt3 is indeed the major ligand produced in epithelial cells. However, the major source of its expression (at the mRNA level) is mainly in secretory progenitor cells, which was confirmed by labeling these cells with the *Defa6-iCre* driver (Fig. 5AB). The fact that no disruption of homeostasis occurs after the loss of Paneth cells in *Tcf7l2*^*flox/flox*^*/Defa6-iCre* mice or after blocking the secretion of Wnt ligands from the producing cells by a conditional *Wls* allele did not surprise us in view of the above (extraepithelial sources of Wnt ligands). Similarly, the lack of an observable phenotype when *Defa6-iCre* and *Pdgfra-CreER*^*T2*^ drivers are used simultaneously can be explained by partial recombination of the cKO alleles and thus partial inactivation of the *Wls* gene. Complete recombination does not occur in organoids either, which could explain the survival of epithelial cells in an in vitro environment, where there are no extraepithelial sources of Wnt ligands. An alternative explanation could be that there are other, probably progenitor cells that can produce the Wnt ligands. For example, after depletion of Paneth cells with DTA, enteroendocrine cells or tuft cells localized at the crypt base can serve as a source of Wnt ligands [114].

The *Defa6-iCre* driver was also used for cell labeling and cellular composition analysis of colorectal tumors. We performed these experiments because the induced deletion of the floxed allele of the tumor suppressor gene *Apc* leads to an apparent increase in the expression of genes typically found in the epithelium of the small intestine. This signature is particularly striking in the time intervals from 24 to 48 h after Apc inactivation and appears to be "masked" in later stages by the appearance of tumor-specific markers [18]. Interestingly, the labeled cells in early tumors in both small intestine and colon did not form contiguous glandular structures but were scattered over different parts of the lesion (Fig. 6AB). At later time intervals, however, we were able to observe fully labeled glandular structures in colon adenomas. These structures gradually increased in size with time elapsing between the appearance of the tumor and its analysis (Fig. 6B). A similar staining pattern was observed in tumors that developed spontaneously in *Apc*^+*/Min*^ mice, i.e., mice that were not treated with AOM and DSS. Remarkably, the mere damage to colon tissue never resulted in cell labeling by the *Defa6-iCre* driver (Fig. 6B). This suggests that the appearance of Defa6-tdTom cells is indeed related to cell transformation and not only to tissue damage. How does this pattern of labeled cells arise? Our hypothesis is that during the formation and growth of a tumor triggered by the loss of Apc, a subset of cells activates a secretory program that results in a “scattered cells” pattern. As the tumor grows, some of these cells may adopt a stem-like phenotype that forms the basis for the glandular structures. The observed process of a (stochastic) reversal of the phenotype of Defa6-tdTom cells from a secretory type (see below), or possibly an earlier activation of *Defa6-iCre* expression in a group of so-called tumor stem cells, is consistent with the fact that the number and size of glandular structures in *Apc*^+*/Min*^ mouse tumors are quite variable, but always correlate with the size (age) of the tumor.

The analysis of the tumor composition using scRNA-seq yielded several interesting results. The comparison of all cells isolated from the tumor with Defa6-tdTom cells showed that both samples contained similar cell clusters, which were represented in comparable proportions. An exception was cluster 3 (secretory progenitor cells), which according to immunohistochemical staining for Muc2 (Fig. 7A) consists of Defa6-tdTom cells distributed throughout the tumor. It appears that these cells make up a substantial part of the tumor in the initial growth phase. When these cells are isolated (sorted) from the tumor, their ability to form organoids is limited (Fig. 7D). In the normal digestive tract, Paneth cells are mainly found in the small intestine. However, in various disease states, they can be abnormally present throughout the digestive tract [115]. The presence of Paneth cells in colorectal adenomas was first documented over fifty years ago [116]. The reported frequencies of the presence of Paneth cells in colorectal adenomas vary considerably, ranging from 0.2% to 39%. Paneth cells are observed more frequently in CRCs than in tubular adenomas [117]. A recent study has shown an association between Paneth cell-containing adenomas, male gender and increased adenoma burden [118]. In addition, recent research has shown that the accumulation of Paneth cells in early colorectal adenomas is related to β-catenin signaling, suggesting that Paneth cells may form a stem cell niche for adenoma cells, which could have therapeutic implications [119].

Several clusters identified by scRNA-seq analysis showed a high level of Wnt signaling activation. This was expected since the tumor process was triggered by the loss of *Apc* and subsequent hyperactivation of Wnt signaling. Interestingly, we were able to follow a developmental trajectory. Most clusters with a "high" Wnt signature were directed towards cluster number 5, which consisted of cells with the highest expression of target genes of the Wnt signaling pathway and the highest proliferation. *Wnt3* was almost exclusively produced in secretory progenitor cells (cluster 3), but it was evident that additional genes encoding Wnt ligands were activated in the terminal cluster 5, particularly the *Wnt6* and *Wnt10b* genes (Fig. 6E). Regarding the signaling pathways involved, Wnt6 has been shown to act via the non-canonical pathway, specifically the planar cell polarity pathway, rather than canonical β-catenin-dependent signaling [120]. This shows that different cell types in the tumor influence each other in this way and can also influence cells in the tumor stroma. Another interesting fact was that the RNA trajectories to cluster 5 contained cells that showed activation of genes regulated by the Hippo signaling pathway. The activation of these genes has been described previously and is particularly associated with intestinal tissue damage in the given model of tumorigenesis [121]. Numerous studies have shown that so-called oncofetal genes regulated by the Hippo pathway (or genes indicative of tissue regeneration after injury) are activated to varying degrees in human CRC or in mouse models of tumorigenesis [122]. The external signals that trigger such activation and their functional significance are not yet known. Our analysis suggests that the Hippo signature is incorporated into the development of tumor cells, and thus has a dynamic (transient) character.

Finally, inactivation of the *Tcf7l2* gene led to significant suppression of the expression profile of the secretory progenitor cells and to reorientation of these cells towards the goblet cell phenotype (Fig. 7B). Thus, the results obtained with the inactivation of *Tcf7l2* in the small intestine could also be observed to a certain extent in colorectal adenomas. Considering the production of the differentiated epithelial cell marker Krt20 (Fig. 7AB), it could be concluded that a method mimicking the suppression of *Tcf7l2* expression (inhibition of the Wnt pathway) could be a therapeutic approach to the treatment of colorectal tumors. Intriguingly, colorectal cancer characterized by a high number of goblet cells represents a distinct subtype with specific molecular features [123] and different developmental mechanisms compared to non-mucinous carcinomas [124]. Mucinous colorectal cancer is often associated with a poorer prognosis than non-mucinous types, particularly in advanced stages [125,126]. A plausible explanation is that the abundant mucin may interfere with the effectiveness of chemotherapy by acting as a barrier to the diffusion of chemotherapeutic drugs [127]. However, the origin and role of goblet cells in colorectal cancer remain unclear and warrant further investigation.

Collectively, our findings suggest that Tcf4 not only regulates secretory cell lineage specification but also modulates tumor cell identity, potentially influencing tumor-microbiome interactions in colorectal cancer.

## Conclusions

This study highlights the critical role of Tcf4 in regulating cell fate in both healthy intestinal epithelium and colon tumors. We demonstrate that partial inactivation of Tcf4 in the small intestinal epithelium leads to the formation of hyperproliferative crypts capable of regenerating the entire tissue. However, the loss of Tcf4 also suppresses antimicrobial peptide expression, particularly α-defensins, leading to microbial dysbiosis and animal mortality before the tissue recovery can occur. Interestingly, this disruption of Wnt signaling redirects secretory differentiation from Paneth cells to goblet cells, a phenomenon also observed in colon tumors.

Our results further reveal distinct subpopulations of tumor cells characterized by differential activation of the Wnt and Hippo signaling pathways. While Hippo signaling is primarily studied in the context of epithelial regeneration, its precise role in intestinal tumorigenesis remains unclear. Additionally, we identified a significant population of Paneth-like secretory tumor cells that produce Wnt ligands, which may influence tumor cell behavior and interactions with the tumor stroma. Overall, the characterization of tumor cell types and their interactions could have significant implications for the diagnosis and prognosis of colorectal cancer.

## Supplementary Information

Supplementary material 1.

Supplementary material 2.

Supplementary material 3.

Supplementary material 4.

Supplementary material 5.

Supplementary material 6.

Supplementary material 7.

Supplementary material 8.

## Data Availability

The data that support the findings of this study are openly available either in the ArrayExpress or in the Sequence Read Archive of the National Center for Biotechnology Information and in the supplementary material of this article. MIAME-compliant (Minimum Information About a Microarray Experiment) RNA-seq data have been deposited in the ArrayExpress database (https://www.ebi.ac.uk/biostudies/arrayexpress) under the following accession numbers: E-MTAB-6915, E-MTAB-13606, E-MTAB-13730, E-MTAB-13731, E-MTAB-13747, E-MTAB-13752, and E-MTAB-14489. Microbiome analysis sequencing data are available in the European Nucleotide Archive (ENA) (https://www.ebi.ac.uk/ena/browser/home) the under accession number PRJEB74647.

## Abbreviations

4-OHT: 4-hydroxytamoxifen
Ace2: angiotensin converting enzyme 2
Agr2: anterior gradient 2
Anxa1: annexin A1
Alpi: alkaline phosphatase
AOM: azozymethane
Apc: adenomatous polyposis coli
Ascl2: achaete-scute family bHLH transcription factor 2
Atoh1: atonal bHLH transcription factor 1
ChgA: chromogranin A
cKO: conditional knockout
cl. Casp3: cleaved caspase 3
Clca1: chloride channel accessory 1
Dclk1: doublecortin-like kinase
DCS: deep crypt secretory
Defa: defensin alpha
Dll1/4: Delta-like canonical Notch ligand 1/4
DSS: sodium dextran sulfate
DTA: diphtheria toxin A
duo: duodenum
ENR: complete organoid culture medium containing Egf, Noggin and Rspondin 1
Fabp1: fatty acid binding protein 1
FACS: fluorescence-activated cell sorting
FC: fold change
Fcgbp: marker Fc gamma binding protein
Fzd: frizzled
Gfra3: glial cell line derived neurotrophic factor family receptor alpha 3
GO: gene ontology
GSEA: Gene Set Enrichment Analysis
Hbegf: heparin binding EGF like growth factor
Hes1: Hes family basic helix-loop-helix (bHLH) transcription factor 1
icGCs: intercrypt goblet cells
Ido1: indoleamine 2,3-dioxygenase 1
ile: ileum
ISCs: intestinal stem cells
jej: jejunum
Krt20: cytokeratin 20
Lef1: lymphoid enhancer-binding factor 1
Lgr4/5: leucine-rich repeat-containing G protein-coupled receptor 4/5
Ly6a: lymphocyte antigen-6
Lyz: lysozyme
Min: multiple intestinal neoplasia
Mki67: proliferation marker Ki-67
Mmp7: matrix metallopeptidase 7
Mptx2: mucosal pentraxin 2
mt: mitochondrial
Muc2: mucin 2
Neurog3: neurogenin 3
Nkd1: naked cuticle homolog 1
Notch1: Notch homolog protein 1
Olfm4: olfactomedin 4
PCA: principal component analysis
Pck1: phosphoenolpyruvate carboxykinase 1
PCNA: proliferating cell nuclear antigen
Rasgef1b: RasGEF domain family member 1b
Reg3b: regenerating islet-derived 3 beta
Reg4: regenerating family member 4
RFP: red fluorescent protein
Rnf43: ring finger protein 43
RT-qPCR: reverse transcription Quantitative polymerase chain reaction
SD: standard deviation
SEM: standard mean of error
Sis: sucrase-isomaltase
Sox9: sex-determining region Y (SRY)-box 9
Spdef: SAM pointed domain containing ETS transcription factor
Spink4: serine peptidase inhibitor, Kazal type 4
TA: transit amplifying
Tacstd2: tumor-associated calcium signal transducer 2
Tcf4: T-cell factor 4
Tcf7l2: transcription factor 7-like 2
Tff3: trefoil factor 3
Tnfrsf19: tumor necrosis factor receptor superfamily, member 19
UMAP: uniform manifold approximation and projection
Wif1: Wnt inhibitory factor 1
Wls: Wntless
wt: wild-type

## References

[1] Tetteh PW, Farin HF, Clevers H. Plasticity within stem cell hierarchies in mammalian epithelia. Trends Cell Biol. 2015;25(2):100–8. 25308311 10.1016/j.tcb.2014.09.003

[2] Tian H, Biehs B, Chiu C, Siebel CW, Wu Y, Costa M, et al. Opposing activities of Notch and Wnt signaling regulate intestinal stem cells and gut homeostasis. Cell Rep. 2015;11(1):33–42. 25818302 10.1016/j.celrep.2015.03.007 PMC4394041

[3] Birchenough GM, Johansson ME, Gustafsson JK, Bergstrom JH, Hansson GC. New developments in goblet cell mucus secretion and function. Mucosal Immunol. 2015;8(4):712–9. 25872481 10.1038/mi.2015.32 PMC4631840

[4] Zhu G, Hu J, Xi R. The cellular niche for intestinal stem cells: a team effort. Cell Regen. 2021;10(1):1. 33385259 10.1186/s13619-020-00061-5 PMC7775856

[5] Sasaki N, Sachs N, Wiebrands K, Ellenbroek SI, Fumagalli A, Lyubimova A, et al. Reg4+ deep crypt secretory cells function as epithelial niche for Lgr5+ stem cells in colon. Proc Natl Acad Sci U S A. 2016;113(37):E5399–407. 27573849 10.1073/pnas.1607327113 PMC5027439

[6] Rothenberg ME, Nusse Y, Kalisky T, Lee JJ, Dalerba P, Scheeren F, et al. Identification of a cKit(+) colonic crypt base secretory cell that supports Lgr5(+) stem cells in mice. Gastroenterology. 2012. 10.1053/j.gastro.2012.02.006 PMC3911891 22333952

[7] Wehkamp J, Wang G, Kubler I, Nuding S, Gregorieff A, Schnabel A, et al. The Paneth cell alpha-defensin deficiency of ileal Crohn’s disease is linked to Wnt/Tcf-4. J Immunol. 2007;179(5):3109–18. 17709525 10.4049/jimmunol.179.5.3109

[8] van Es JH, Haegebarth A, Kujala P, Itzkovitz S, Koo BK, Boj SF, et al. A critical role for the wnt effector Tcf4 in Adult Intestinal Homeostatic Self-Renewal. Mol Cell Biol. 2012. 10.1128/MCB.06288-11 PMC3347420 22393260

[9] Korinek V, Barker N, Moerer P, van Donselaar E, Huls G, Peters PJ, et al. Depletion of epithelial stem-cell compartments in the small intestine of mice lacking Tcf-4. Nat Genet. 1998;19(4):379–83. 9697701 10.1038/1270

[10] Hrckulak D, Janeckova L, Lanikova L, Kriz V, Horazna M, Babosova O, et al. Wnt effector TCF4 Is dispensable for Wnt signaling in human cancer cells. Genes (Basel). 2018;9(9). 10.3390/genes9090439 PMC6162433 30200414

[11] Farin HF, Van Es JH, Clevers H. Redundant sources of Wnt regulate intestinal stem cells and promote formation of paneth cells. Gastroenterology. 2012. 10.1053/j.gastro.2012.08.031 22922422

[12] Sato T, Vries RG, Snippert HJ, van de Wetering M, Barker N, Stange DE, et al. Single Lgr5 stem cells build crypt-villus structures in vitro without a mesenchymal niche. Nature. 2009;459(7244):262–5. 19329995 10.1038/nature07935

[13] Valenta T, Degirmenci B, Moor AE, Herr P, Zimmerli D, Moor MB, et al. Wnt ligands secreted by subepithelial mesenchymal cells are essential for the survival of intestinal stem cells and gut homeostasis. Cell Rep. 2016;15(5):911–8. 27117411 10.1016/j.celrep.2016.03.088

[14] Degirmenci B, Valenta T, Dimitrieva S, Hausmann G, Basler K. GLI1-expressing mesenchymal cells form the essential Wnt-secreting niche for colon stem cells. Nature. 2018;558(7710):449–53. 29875413 10.1038/s41586-018-0190-3

[15] Clevers H, Nusse R. Wnt/beta-catenin signaling and disease. Cell. 2012;149(6):1192–205. 22682243 10.1016/j.cell.2012.05.012

[16] Krausova M, Korinek V. Wnt signaling in adult intestinal stem cells and cancer. Cell Signal. 2014;26(3):570–9. 24308963 10.1016/j.cellsig.2013.11.032

[17] Korinek V, Barker N, Morin PJ, van Wichen D, de Weger R, Kinzler KW, et al. Constitutive transcriptional activation by a beta-catenin-Tcf complex in APC-/- colon carcinoma [see comments]. Science. 1997;275(5307):1784–7. 9065401 10.1126/science.275.5307.1784

[18] Horazna M, Janeckova L, Svec J, Babosova O, Hrckulak D, Vojtechova M, et al. Msx1 loss suppresses formation of the ectopic crypts developed in the Apc-deficient small intestinal epithelium. Sci Rep. 2019;9(1):1629. 30733598 10.1038/s41598-018-38310-y PMC6367488

[19] Su LK, Kinzler KW, Vogelstein B, Preisinger AC, Moser AR, Luongo C, et al. Multiple intestinal neoplasia caused by a mutation in the murine homolog of the APC gene. Science. 1992;256(5057):668–70. 1350108 10.1126/science.1350108

[20] Parang B, Barrett CW, Williams CS. AOM/DSS model of colitis-associated cancer. Methods Mol Biol. 2016;1422:297–307. 27246042 10.1007/978-1-4939-3603-8_26 PMC5035391

[21] Basak O, van de Born M, Korving J, Beumer J, van der Elst S, van Es JH, et al. Mapping early fate determination in Lgr5+ crypt stem cells using a novel Ki67-RFP allele. EMBO J. 2014;33(18):2057–68. 25092767 10.15252/embj.201488017 PMC4195772

[22] Chung MI, Bujnis M, Barkauskas CE, Kobayashi Y, Hogan BLM. Niche-mediated BMP/SMAD signaling regulates lung alveolar stem cell proliferation and differentiation. Development. 2018;145(9). 10.1242/dev.163014 PMC5992594 29752282

[23] Madisen L, Zwingman TA, Sunkin SM, Oh SW, Zariwala HA, Gu H, et al. A robust and high-throughput Cre reporting and characterization system for the whole mouse brain. Nat Neurosci. 2010;13(1):133–40. 20023653 10.1038/nn.2467 PMC2840225

[24] Ventura A, Kirsch DG, McLaughlin ME, Tuveson DA, Grimm J, Lintault L, et al. Restoration of p53 function leads to tumour regression in vivo. Nature. 2007;445(7128):661–5. 17251932 10.1038/nature05541

[25] Voehringer D, Liang HE, Locksley RM. Homeostasis and effector function of lymphopenia-induced “memory-like” T cells in constitutively T cell-depleted mice. J Immunol. 2008;180(7):4742–53. 18354198 10.4049/jimmunol.180.7.4742 PMC2670614

[26] el Marjou F, Janssen KP, Chang BH, Li M, Hindie V, Chan L, et al. Tissue-specific and inducible Cre-mediated recombination in the gut epithelium. Genesis. 2004;39(3):186–93. 15282745 10.1002/gene.20042

[27] Adolph TE, Tomczak MF, Niederreiter L, Ko HJ, Bock J, Martinez-Naves E, et al. Paneth cells as a site of origin for intestinal inflammation. Nature. 2013;503(7475):272–6. 24089213 10.1038/nature12599 PMC3862182

[28] Sato T, Stange DE, Ferrante M, Vries RG, Van Es JH, Van den Brink S, et al. Long-term expansion of epithelial organoids from human colon, adenoma, adenocarcinoma, and Barrett’s epithelium. Gastroenterology. 2011;141(5):1762–72. 21889923 10.1053/j.gastro.2011.07.050

[29] Ootani A, Li X, Sangiorgi E, Ho QT, Ueno H, Toda S, et al. Sustained in vitro intestinal epithelial culture within a Wnt-dependent stem cell niche. Nat Med. 2009;15(6):701–6. 19398967 10.1038/nm.1951 PMC2919216

[30] de Lau W, Barker N, Low TY, Koo BK, Li VS, Teunissen H, et al. Lgr5 homologues associate with Wnt receptors and mediate R-spondin signalling. Nature. 2011;476(7360):293–7. 21727895 10.1038/nature10337

[31] Schindelin J, Arganda-Carreras I, Frise E, Kaynig V, Longair M, Pietzsch T, et al. Fiji: an open-source platform for biological-image analysis. Nat Methods. 2012;9(7):676–82. 22743772 10.1038/nmeth.2019 PMC3855844

[32] Ewels PA, Peltzer A, Fillinger S, Patel H, Alneberg J, Wilm A, et al. The nf-core framework for community-curated bioinformatics pipelines. Nat Biotechnol. 2020;38(3):276–8. 32055031 10.1038/s41587-020-0439-x

[33] Howe KL, Achuthan P, Allen J, Allen J, Alvarez-Jarreta J, Amode MR, et al. Ensembl 2021. Nucleic Acids Res. 2021;49(D1):D884–91. 33137190 10.1093/nar/gkaa942 PMC7778975

[34] Kim D, Paggi JM, Park C, Bennett C, Salzberg SL. Graph-based genome alignment and genotyping with HISAT2 and HISAT-genotype. Nat Biotechnol. 2019;37(8):907–15. 31375807 10.1038/s41587-019-0201-4 PMC7605509

[35] Liao Y, Smyth GK, Shi W. featureCounts: an efficient general purpose program for assigning sequence reads to genomic features. Bioinformatics. 2014;30(7):923–30. 24227677 10.1093/bioinformatics/btt656

[36] Love MI, Huber W, Anders S. Moderated estimation of fold change and dispersion for RNA-seq data with DESeq2. Genome Biol. 2014;15(12):550. 25516281 10.1186/s13059-014-0550-8 PMC4302049

[37] Dobin A, Davis CA, Schlesinger F, Drenkow J, Zaleski C, Jha S, et al. STAR: ultrafast universal RNA-seq aligner. Bioinformatics. 2013;29(1):15–21. 23104886 10.1093/bioinformatics/bts635 PMC3530905

[38] Patro R, Duggal G, Love MI, Irizarry RA, Kingsford C. Salmon provides fast and bias-aware quantification of transcript expression. Nat Methods. 2017;14(4):417–9. 28263959 10.1038/nmeth.4197 PMC5600148

[39] Wu JQ, Mao LB, Liu LF, Li YM, Wu J, Yao J, et al. Identification of key genes and pathways of BMP-9-induced osteogenic differentiation of mesenchymal stem cells by integrated bioinformatics analysis. J Orthop Surg Res. 2021;16(1):273. 33879213 10.1186/s13018-021-02390-w PMC8059242

[40] Chen EY, Tan CM, Kou Y, Duan Q, Wang Z, Meirelles GV, et al. Enrichr: interactive and collaborative HTML5 gene list enrichment analysis tool. BMC Bioinformatics. 2013;14:128. 23586463 10.1186/1471-2105-14-128 PMC3637064

[41] Kuleshov MV, Jones MR, Rouillard AD, Fernandez NF, Duan Q, Wang Z, et al. Enrichr: a comprehensive gene set enrichment analysis web server 2016 update. Nucleic Acids Res. 2016;44(W1):W90–7. 27141961 10.1093/nar/gkw377 PMC4987924

[42] Stuart T, Butler A, Hoffman P, Hafemeister C, Papalexi E, Mauck WM 3rd, et al. Comprehensive integration of single-cell data. Cell. 2019;177(7):1888–902. 31178118 10.1016/j.cell.2019.05.031 PMC6687398

[43] Osorio D, Cai JJ. Systematic determination of the mitochondrial proportion in human and mice tissues for single-cell RNA-sequencing data quality control. Bioinformatics. 2021;37(7):963–7. 32840568 10.1093/bioinformatics/btaa751 PMC8599307

[44] Ruan H, Wang Z, Sun Z, Wei J, Zhang L, Ju H, et al. Single-cell RNA sequencing reveals the characteristics of cerebrospinal fluid tumour environment in breast cancer and lung cancer leptomeningeal metastases. Clin Transl Med. 2022;12(6): e885. 35678121 10.1002/ctm2.885 PMC9178395

[45] Becht E, McInnes L, Healy J, Dutertre CA, Kwok IWH, Ng LG, et al. Dimensionality reduction for visualizing single-cell data using UMAP. Nat Biotechnol. 2018. 10.1038/nbt.4314 30531897

[46] La Manno G, Soldatov R, Zeisel A, Braun E, Hochgerner H, Petukhov V, et al. RNA velocity of single cells. Nature. 2018;560(7719):494–8. 30089906 10.1038/s41586-018-0414-6 PMC6130801

[47] Bendova B, Mikula O, Voslajerova Bimova B, Cizkova D, Daniszova K, Dureje L, et al. Divergent gut microbiota in two closely related house mouse subspecies under common garden conditions. FEMS Microbiol Ecol. 2022;98(8). 10.1093/femsec/fiac078 35767862

[48] Jiang H, Lei R, Ding SW, Zhu S. Skewer: a fast and accurate adapter trimmer for next-generation sequencing paired-end reads. BMC Bioinformatics. 2014;15:182. 24925680 10.1186/1471-2105-15-182 PMC4074385

[49] Callahan BJ, McMurdie PJ, Rosen MJ, Han AW, Johnson AJ, Holmes SP. DADA2: High-resolution sample inference from Illumina amplicon data. Nat Methods. 2016;13(7):581–3. 27214047 10.1038/nmeth.3869 PMC4927377

[50] Edgar RC, Haas BJ, Clemente JC, Quince C, Knight R. UCHIME improves sensitivity and speed of chimera detection. Bioinformatics. 2011;27(16):2194–200. 21700674 10.1093/bioinformatics/btr381 PMC3150044

[51] Quast C, Pruesse E, Yilmaz P, Gerken J, Schweer T, Yarza P, et al. The SILVA ribosomal RNA gene database project: improved data processing and web-based tools. Nucleic Acids Res. 2013;41(1):D590-6. 23193283 10.1093/nar/gks1219 PMC3531112

[52] Wang Q, Garrity GM, Tiedje JM, Cole JR. Naive Bayesian classifier for rapid assignment of rRNA sequences into the new bacterial taxonomy. Appl Environ Microbiol. 2007;73(16):5261–7. 17586664 10.1128/AEM.00062-07 PMC1950982

[53] McMurdie PJ, Holmes S. phyloseq: an R package for reproducible interactive analysis and graphics of microbiome census data. PLoS ONE. 2013;8(4): e61217. 23630581 10.1371/journal.pone.0061217 PMC3632530

[54] Barnett DJ, Arts IC, Penders J. microViz: an R package for microbiome data visualization and statistics. J Open Source Softw. 2021;6(63):4.

[55] Brandl K, Plitas G, Schnabl B, DeMatteo RP, Pamer EG. MyD88-mediated signals induce the bactericidal lectin RegIII gamma and protect mice against intestinal Listeria monocytogenes infection. J Exp Med. 2007;204(8):1891–900. 17635956 10.1084/jem.20070563 PMC2118673

[56] Vaishnava S, Yamamoto M, Severson KM, Ruhn KA, Yu X, Koren O, et al. The antibacterial lectin RegIIIgamma promotes the spatial segregation of microbiota and host in the intestine. Science. 2011;334(6053):255–8. 21998396 10.1126/science.1209791 PMC3321924

[57] Lomasney KW, Cryan JF, Hyland NP. Converging effects of a Bifidobacterium and Lactobacillus probiotic strain on mouse intestinal physiology. Am J Physiol Gastrointest Liver Physiol. 2014;307(2):G241–7. 24852567 10.1152/ajpgi.00401.2013

[58] Zhang Y, Tu S, Ji X, Wu J, Meng J, Gao J, et al. Dubosiella newyorkensis modulates immune tolerance in colitis via the L-lysine-activated AhR-IDO1-Kyn pathway. Nat Commun. 2024;15(1):1333. 38351003 10.1038/s41467-024-45636-x PMC10864277

[59] Thorkildsen LT, Nwosu FC, Avershina E, Ricanek P, Perminow G, Brackmann S, et al. Dominant fecal microbiota in newly diagnosed untreated inflammatory bowel disease patients. Gastroenterol Res Pract. 2013;2013: 636785. 24348539 10.1155/2013/636785 PMC3855989

[60] Barker N, van Es JH, Kuipers J, Kujala P, van den Born M, Cozijnsen M, et al. Identification of stem cells in small intestine and colon by marker gene Lgr5. Nature. 2007;449(7165):1003–7. 17934449 10.1038/nature06196

[61] van der Flier LG, Haegebarth A, Stange DE, van de Wetering M, Clevers H. OLFM4 is a robust marker for stem cells in human intestine and marks a subset of colorectal cancer cells. Gastroenterology. 2009;137(1):15–7. 19450592 10.1053/j.gastro.2009.05.035

[62] Buczacki SJ, Zecchini HI, Nicholson AM, Russell R, Vermeulen L, Kemp R, et al. Intestinal label-retaining cells are secretory precursors expressing Lgr5. Nature. 2013;495(7439):65–9. 23446353 10.1038/nature11965

[63] Franzen O, Gan LM, Bjorkegren JLM. PanglaoDB: a web server for exploration of mouse and human single-cell RNA sequencing data. Database (Oxford). 2019;2019. 10.1093/database/baz046 PMC6450036 30951143

[64] Norkin M, Capdevila C, Calderon RI, Su T, Trifas M, Ordonez-Moran P, et al. Single-cell studies of intestinal stem cell heterogeneity during homeostasis and regeneration. Methods Mol Biol. 2020;2171:155–67. 32705640 10.1007/978-1-0716-0747-3_9

[65] Yang Q, Bermingham NA, Finegold MJ, Zoghbi HY. Requirement of Math1 for secretory cell lineage commitment in the mouse intestine. Science. 2001;294(5549):2155–8. 11739954 10.1126/science.1065718

[66] Lo YH, Chung E, Li Z, Wan YW, Mahe MM, Chen MS, et al. Transcriptional regulation by ATOH1 and its target SPDEF in the intestine. Cell Mol Gastroenterol Hepatol. 2017;3(1):51–71. 28174757 10.1016/j.jcmgh.2016.10.001 PMC5247424

[67] Bjerknes M, Cheng H. Neurogenin 3 and the enteroendocrine cell lineage in the adult mouse small intestinal epithelium. Dev Biol. 2006;300(2):722–35. 17007831 10.1016/j.ydbio.2006.07.040

[68] Drokhlyansky E, Smillie CS, VanWittenberghe N, Ericsson M, Griffin GK, Eraslan G, et al. The human and mouse enteric nervous system at single-cell resolution. Cell. 2020;182(6):1606–22. 10.1016/j.cell.2020.08.003 PMC8358727 32888429

[69] Bottcher A, Buttner M, Tritschler S, Sterr M, Aliluev A, Oppenlander L, et al. Non-canonical Wnt/PCP signalling regulates intestinal stem cell lineage priming towards enteroendocrine and Paneth cell fates. Nat Cell Biol. 2021;23(1):23–31. 33398177 10.1038/s41556-020-00617-2

[70] Malmsten M, Davoudi M, Walse B, Rydengard V, Pasupuleti M, Morgelin M, et al. Antimicrobial peptides derived from growth factors. Growth Factors. 2007;25(1):60–70. 17454151 10.1080/08977190701344120

[71] Fan Z, Peng W, Wang Z, Zhang L, Liu K. Identification of biomarkers associated with metabolic cardiovascular disease using mRNA-SNP-miRNA regulatory network analysis. BMC Cardiovasc Disord. 2021;21(1):351. 34301176 10.1186/s12872-021-02166-4 PMC8305867

[72] Leao FB, Vaughn LS, Bhatt D, Liao W, Maloney D, Carvalho BC, et al. Toll-like receptor (TLR)-induced Rasgef1b expression in macrophages is regulated by NF-kappaB through its proximal promoter. Int J Biochem Cell Biol. 2020;127: 105840. 32866686 10.1016/j.biocel.2020.105840

[73] Borrelli C, Valenta T, Handler K, Velez K, Gurtner A, Moro G, et al. Differential regulation of beta-catenin-mediated transcription via N- and C-terminal co-factors governs identity of murine intestinal epithelial stem cells. Nat Commun. 2021;12(1):1368. 33649334 10.1038/s41467-021-21591-9 PMC7921392

[74] Sato T, van Es JH, Snippert HJ, Stange DE, Vries RG, van den Born M, et al. Paneth cells constitute the niche for Lgr5 stem cells in intestinal crypts. Nature. 2011;469(7330):415–8. 21113151 10.1038/nature09637 PMC3547360

[75] Fafilek B, Krausova M, Vojtechova M, Pospichalova V, Tumova L, Sloncova E, et al. Troy, a tumor necrosis factor receptor family member, interacts with lgr5 to inhibit wnt signaling in intestinal stem cells. Gastroenterology. 2013;144(2):381–91. 23142137 10.1053/j.gastro.2012.10.048

[76] Song C, Chai Z, Chen S, Zhang H, Zhang X, Zhou Y. Intestinal mucus components and secretion mechanisms: what we do and do not know. Exp Mol Med. 2023;55(4):681–91. 37009791 10.1038/s12276-023-00960-y PMC10167328

[77] Bosdriesz E, Molenaar D, Teusink B, Bruggeman FJ. How fast-growing bacteria robustly tune their ribosome concentration to approximate growth-rate maximization. FEBS J. 2015;282(10):2029–44. 25754869 10.1111/febs.13258 PMC4672707

[78] Shore D, Zencir S, Albert B. Transcriptional control of ribosome biogenesis in yeast: links to growth and stress signals. Biochem Soc Trans. 2021;49(4):1589–99. 34240738 10.1042/BST20201136 PMC8421047

[79] Huggins IJ, Bos T, Gaylord O, Jessen C, Lonquich B, Puranen A, et al. The WNT target SP5 negatively regulates WNT transcriptional programs in human pluripotent stem cells. Nat Commun. 2017;8(1):1034. 29044119 10.1038/s41467-017-01203-1 PMC5647328

[80] van der Flier LG, van Gijn ME, Hatzis P, Kujala P, Haegebarth A, Stange DE, et al. Transcription factor achaete scute-like 2 controls intestinal stem cell fate. Cell. 2009;136(5):903–12. 19269367 10.1016/j.cell.2009.01.031

[81] Mori-Akiyama Y, van den Born M, van Es JH, Hamilton SR, Adams HP, Zhang J, et al. SOX9 is required for the differentiation of paneth cells in the intestinal epithelium. Gastroenterology. 2007;133(2):539–46. 17681175 10.1053/j.gastro.2007.05.020

[82] Asfaha S, Hayakawa Y, Muley A, Stokes S, Graham TA, Ericksen RE, et al. Krt19(+)/Lgr5(-) cells are radioresistant cancer-initiating stem cells in the colon and intestine. Cell Stem Cell. 2015;16(6):627–38. 26046762 10.1016/j.stem.2015.04.013 PMC4457942

[83] Nystrom EEL, Martinez-Abad B, Arike L, Birchenough GMH, Nonnecke EB, Castillo PA, et al. An intercrypt subpopulation of goblet cells is essential for colonic mucus barrier function. Science. 2021;372(6539):eabb1590. 33859001 10.1126/science.abb1590 PMC8542866

[84] Mustata RC, Van Loy T, Lefort A, Libert F, Strollo S, Vassart G, et al. Lgr4 is required for Paneth cell differentiation and maintenance of intestinal stem cells ex vivo. EMBO Rep. 2011;12(6):558–64. 21508962 10.1038/embor.2011.52 PMC3128273

[85] Luo K, Cao SS. Endoplasmic reticulum stress in intestinal epithelial cell function and inflammatory bowel disease. Gastroenterol Res Pract. 2015;2015: 328791. 25755668 10.1155/2015/328791 PMC4338396

[86] van Es JH, van Gijn ME, Riccio O, van den Born M, Vooijs M, Begthel H, et al. Notch/gamma-secretase inhibition turns proliferative cells in intestinal crypts and adenomas into goblet cells. Nature. 2005;435(7044):959–63. 15959515 10.1038/nature03659

[87] Koo BK, Spit M, Jordens I, Low TY, Stange DE, van de Wetering M, et al. Tumour suppressor RNF43 is a stem-cell E3 ligase that induces endocytosis of Wnt receptors. Nature. 2012;488(7413):665–9. 22895187 10.1038/nature11308

[88] Wang Z, Shu W, Lu MM, Morrisey EE. Wnt7b activates canonical signaling in epithelial and vascular smooth muscle cells through interactions with Fzd1, Fzd10, and LRP5. Mol Cell Biol. 2005;25(12):5022–30. 15923619 10.1128/MCB.25.12.5022-5030.2005 PMC1140585

[89] Ouko L, Ziegler TR, Gu LH, Eisenberg LM, Yang VW. Wnt11 signaling promotes proliferation, transformation, and migration of IEC6 intestinal epithelial cells. J Biol Chem. 2004;279(25):26707–15. 15084607 10.1074/jbc.M402877200 PMC1351009

[90] Nishioka M, Ueno K, Hazama S, Okada T, Sakai K, Suehiro Y, et al. Possible involvement of Wnt11 in colorectal cancer progression. Mol Carcinog. 2013;52(3):207–17. 22161723 10.1002/mc.21845

[91] Durand A, Donahue B, Peignon G, Letourneur F, Cagnard N, Slomianny C, et al. Functional intestinal stem cells after Paneth cell ablation induced by the loss of transcription factor Math1 (Atoh1). Proc Natl Acad Sci U S A. 2012;109(23):8965–70. 22586121 10.1073/pnas.1201652109 PMC3384132

[92] Gay MH, Valenta T, Herr P, Paratore-Hari L, Basler K, Sommer L. Distinct adhesion-independent functions of beta-catenin control stage-specific sensory neurogenesis and proliferation. BMC Biol. 2015;13:24. 25885041 10.1186/s12915-015-0134-4 PMC4416270

[93] Greicius G, Kabiri Z, Sigmundsson K, Liang C, Bunte R, Singh MK, et al. PDGFRalpha(+) pericryptal stromal cells are the critical source of Wnts and RSPO3 for murine intestinal stem cells in vivo. Proc Natl Acad Sci U S A. 2018;115(14):E3173–81. 29559533 10.1073/pnas.1713510115 PMC5889626

[94] Brugger MD, Valenta T, Fazilaty H, Hausmann G, Basler K. Distinct populations of crypt-associated fibroblasts act as signaling hubs to control colon homeostasis. PLoS Biol. 2020;18(12): e3001032. 33306673 10.1371/journal.pbio.3001032 PMC7758045

[95] Haber AL, Biton M, Rogel N, Herbst RH, Shekhar K, Smillie C, et al. A single-cell survey of the small intestinal epithelium. Nature. 2017;551(7680):333–9. 29144463 10.1038/nature24489 PMC6022292

[96] Parikh K, Antanaviciute A, Fawkner-Corbett D, Jagielowicz M, Aulicino A, Lagerholm C, et al. Colonic epithelial cell diversity in health and inflammatory bowel disease. Nature. 2019;567(7746):49–55. 30814735 10.1038/s41586-019-0992-y

[97] Burclaff J, Bliton RJ, Breau KA, Ok MT, Gomez-Martinez I, Ranek JS, et al. A proximal-to-distal survey of healthy adult human small intestine and colon epithelium by single-cell transcriptomics. Cell Mol Gastroenterol Hepatol. 2022;13(5):1554–89. 35176508 10.1016/j.jcmgh.2022.02.007 PMC9043569

[98] Matheu A, Collado M, Wise C, Manterola L, Cekaite L, Tye AJ, et al. Oncogenicity of the developmental transcription factor Sox9. Cancer Res. 2012;72(5):1301–15. 22246670 10.1158/0008-5472.CAN-11-3660 PMC3378515

[99] Lu B, Fang Y, Xu J, Wang L, Xu F, Xu E, et al. Analysis of SOX9 expression in colorectal cancer. Am J Clin Pathol. 2008;130(6):897–904. 19019766 10.1309/AJCPW1W8GJBQGCNI

[100] Stastna M, Janeckova L, Hrckulak D, Kriz V, Korinek V. Human colorectal cancer from the perspective of mouse models. Genes (Basel). 2019;10(10):788. 31614493 10.3390/genes10100788 PMC6826908

[101] Stancikova J, Krausova M, Kolar M, Fafilek B, Svec J, Sedlacek R, et al. NKD1 marks intestinal and liver tumors linked to aberrant Wnt signaling. Cell Signal. 2015;27(2):245–56. 25446263 10.1016/j.cellsig.2014.11.008

[102] Hovanes K, Li TW, Munguia JE, Truong T, Milovanovic T, Lawrence Marsh J, et al. Beta-catenin-sensitive isoforms of lymphoid enhancer factor-1 are selectively expressed in colon cancer. Nat Genet. 2001;28(1):53–7. 11326276 10.1038/ng0501-53

[103] Chen L, Qiu X, Dupre A, Pellon-Cardenas O, Fan X, Xu X, et al. TGFB1 induces fetal reprogramming and enhances intestinal regeneration. Cell Stem Cell. 2023;30(11):1520–37. 37865088 10.1016/j.stem.2023.09.015 PMC10841757

[104] Gregorieff A, Liu Y, Inanlou MR, Khomchuk Y, Wrana JL. Yap-dependent reprogramming of Lgr5(+) stem cells drives intestinal regeneration and cancer. Nature. 2015;526(7575):715–8. 26503053 10.1038/nature15382

[105] Yui S, Azzolin L, Maimets M, Pedersen MT, Fordham RP, Hansen SL, et al. YAP/TAZ-dependent reprogramming of colonic epithelium links ECM remodeling to tissue regeneration. Cell Stem Cell. 2018;22(1):35–49. 29249464 10.1016/j.stem.2017.11.001 PMC5766831

[106] Zhu Y, Li X. Advances of Wnt signalling pathway in colorectal cancer. Cells. 2023;12(3):447. 36766788 10.3390/cells12030447 PMC9913588

[107] Middelhoff M, Westphalen CB, Hayakawa Y, Yan KS, Gershon MD, Wang TC, et al. Dclk1-expressing tuft cells: critical modulators of the intestinal niche? Am J Physiol Gastrointest Liver Physiol. 2017;313(4):G285–99. 28684459 10.1152/ajpgi.00073.2017 PMC5668570

[108] Noah TK, Kazanjian A, Whitsett J, Shroyer NF. SAM pointed domain ETS factor (SPDEF) regulates terminal differentiation and maturation of intestinal goblet cells. Exp Cell Res. 2010;316(3):452–65. 19786015 10.1016/j.yexcr.2009.09.020 PMC3004755

[109] Chen L, Toke NH, Luo S, Vasoya RP, Fullem RL, Parthasarathy A, et al. A reinforcing HNF4-SMAD4 feed-forward module stabilizes enterocyte identity. Nat Genet. 2019;51(5):777–85. 30988513 10.1038/s41588-019-0384-0 PMC6650150

[110] Grun D, Lyubimova A, Kester L, Wiebrands K, Basak O, Sasaki N, et al. Single-cell messenger RNA sequencing reveals rare intestinal cell types. Nature. 2015;525(7568):251–5. 26287467 10.1038/nature14966

[111] Kuhnert F, Davis CR, Wang HT, Chu P, Lee M, Yuan J, et al. Essential requirement for Wnt signaling in proliferation of adult small intestine and colon revealed by adenoviral expression of Dickkopf-1. Proc Natl Acad Sci U S A. 2004;101(1):266–71. 14695885 10.1073/pnas.2536800100 PMC314174

[112] Hilkens J, Timmer NC, Boer M, Ikink GJ, Schewe M, Sacchetti A, et al. RSPO3 expands intestinal stem cell and niche compartments and drives tumorigenesis. Gut. 2017;66(6):1095–105. 27511199 10.1136/gutjnl-2016-311606 PMC5532462

[113] Zhou JY, Zan GX, Zhu QJ, Gao CQ, Yan HC, Wang XQ. Recombinant porcine R-Spondin 1 facilitates intestinal stem cell expansion along the crypt-villus axis through potentiating Wnt/beta-catenin signaling in homeostasis and deoxynivalenol injury. J Agric Food Chem. 2022;70(34):10644–53. 35997221 10.1021/acs.jafc.2c02013

[114] van Es JH, Wiebrands K, Lopez-Iglesias C, van de Wetering M, Zeinstra L, van den Born M, et al. Enteroendocrine and tuft cells support Lgr5 stem cells on Paneth cell depletion. Proc Natl Acad Sci U S A. 2019;116(52):26599–605. 31843916 10.1073/pnas.1801888117 PMC6936398

[115] Nihed A, Soumaya M, Atika B, Ilhem BJ, Atef BA, Fehmi H, et al. Paneth cell adenocarcinoma of the colon: a rare entity. Int J Surg Case Rep. 2019;65:313–6. 31766010 10.1016/j.ijscr.2019.10.071 PMC6881596

[116] Gibbs NM. Incidence and significance of argentaffin and paneth cells in some tumours of the large intestine. J Clin Pathol. 1967;20(6):826–31. 5614067 10.1136/jcp.20.6.826 PMC473612

[117] Mahon M, Xu J, Yi X, Liu X, Gao N, Zhang L. Paneth cell in adenomas of the distal colorectum is inversely associated with synchronous advanced adenoma and carcinoma. Sci Rep. 2016;6:26129. 27188450 10.1038/srep26129 PMC4870568

[118] Pai RK, Rybicki LA, Goldblum JR, Shen B, Xiao SY, Liu X. Paneth cells in colonic adenomas: association with male sex and adenoma burden. Am J Surg Pathol. 2013;37(1):98–103. 23232853 10.1097/PAS.0b013e318267b02e

[119] Lopez-Arribillaga E, Yan B, Lobo-Jarne T, Guillen Y, Menendez S, Andreu M, et al. Accumulation of paneth cells in early colorectal adenomas is associated with beta-catenin signaling and poor patient prognosis. Cells. 2021;10(11):2928. 34831152 10.3390/cells10112928 PMC8616107

[120] Zhan T, Rindtorff N, Boutros M. Wnt signaling in cancer. Oncogene. 2017;36(11):1461–73. 27617575 10.1038/onc.2016.304 PMC5357762

[121] Cai J, Zhang N, Zheng Y, de Wilde RF, Maitra A, Pan D. The Hippo signaling pathway restricts the oncogenic potential of an intestinal regeneration program. Genes Dev. 2010;24(21):2383–8. 21041407 10.1101/gad.1978810 PMC2964748

[122] Sharma A, Bleriot C, Currenti J, Ginhoux F. Oncofetal reprogramming in tumour development and progression. Nat Rev Cancer. 2022;22(10):593–602. 35999292 10.1038/s41568-022-00497-8

[123] Miller SA, Ghobashi AH, O’Hagan HM. Consensus molecular subtyping of colorectal cancers is influenced by goblet cell content. Cancer Genet. 2021;254–255:34–9. 33571895 10.1016/j.cancergen.2021.01.009

[124] Jesinghaus M, Konukiewitz B, Foersch S, Stenzinger A, Steiger K, Muckenhuber A, et al. Appendiceal goblet cell carcinoids and adenocarcinomas ex-goblet cell carcinoid are genetically distinct from primary colorectal-type adenocarcinoma of the appendix. Mod Pathol. 2018;31(5):829–39. 29327707 10.1038/modpathol.2017.184

[125] Fadel MG, Malietzis G, Constantinides V, Pellino G, Tekkis P, Kontovounisios C. Clinicopathological factors and survival outcomes of signet-ring cell and mucinous carcinoma versus adenocarcinoma of the colon and rectum: a systematic review and meta-analysis. Discov Oncol. 2021;12(1):5. 35201441 10.1007/s12672-021-00398-6 PMC8762524

[126] Luo C, Cen S, Ying J, Wang X, Fu Z, Liu P, et al. Tumor clinicopathological characteristics and their prognostic value in mucinous colorectal carcinoma. Future Oncol. 2019;15(35):4095–104. 31773976 10.2217/fon-2019-0342

[127] Jonckheere N, Skrypek N, Van Seuningen I. Mucins and tumor resistance to chemotherapeutic drugs. Biochim Biophys Acta. 2014;1846(1):142–51. 24785432 10.1016/j.bbcan.2014.04.008

