# Supplementary material for: Tcf4 regulates secretory cell fate decisions in the small intestine and colon tumors: insights from transcriptomic, histological, and microbiome analyses
Source: Stem Cell Res Ther. 2025 Apr 12;16:170. doi: 10.1186/s13287-025-04280-y (PMC11993999; doi:10.1186/s13287-025-04280-y)
Supplement: Supplementary file 8 — Supplementary material 8. [file 13287_2025_4280_MOESM8_ESM.docx]

**SUPPLEMENTARY MATERIAL**

**
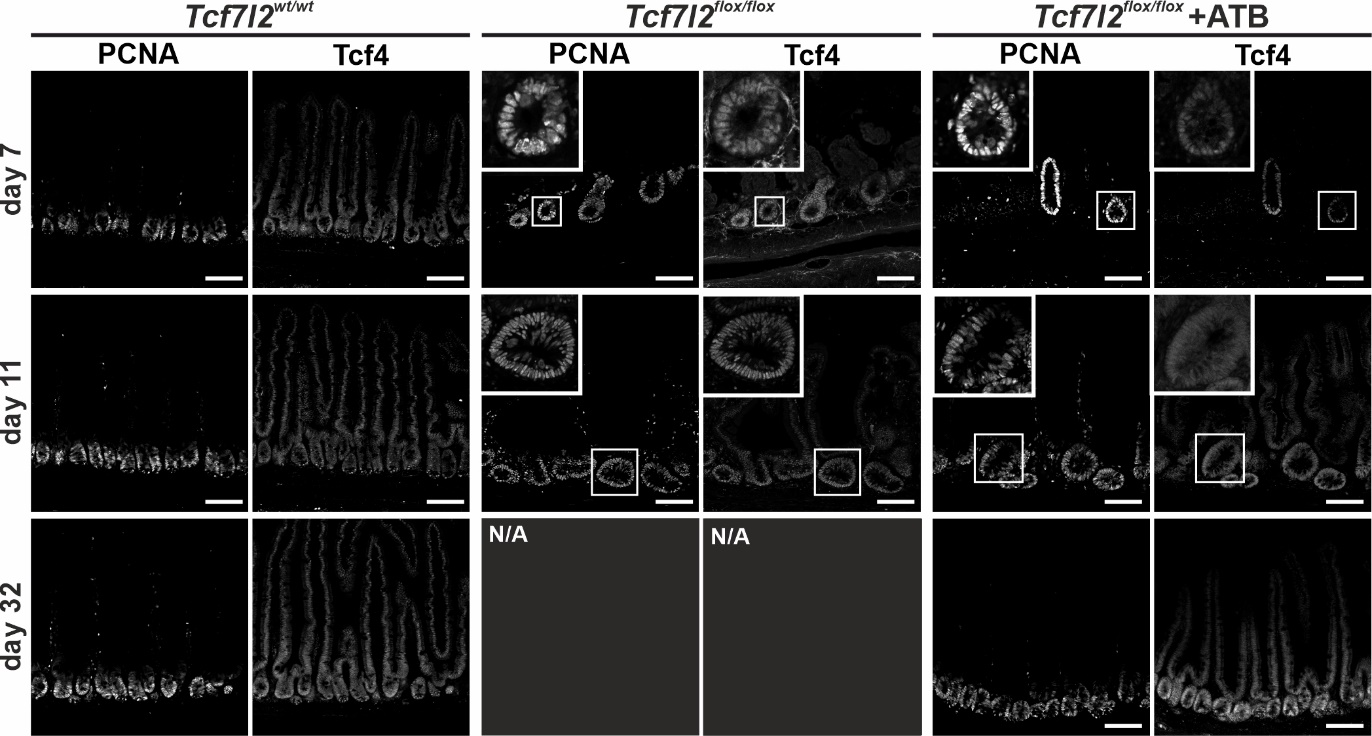
Supplementary Figure and Table Legends**

**Supplementary Figure S1. Analysis of the effects of Tcf4 inactivation in the small intestinal epithelium.** Grayscale version of the photomicrographs shown in Fig. 1A. Immunohistochemical staining of T-cell factor 4 (Tcf4, green) and proliferating cell nuclear antigen (PCNA, red) protein localization in the small intestine of wild-type *Tcf7l2^wt/wt^/VillinCreER^T2^* (*Tcf7l2^wt/wt^*) and knockout *Tcf7l2^flox/flox^ /VillinCreER^T2^* (*Tcf7l2^flox/flox^*) mice, with antibiotics (ATB) and without antibiotic treatment, over time after tamoxifen administration. The insets show increased magnification of the framed areas. Scale bar: 50 μm. Tcf7l2, transcription factor 7 like 2; wt, wild-type.

**
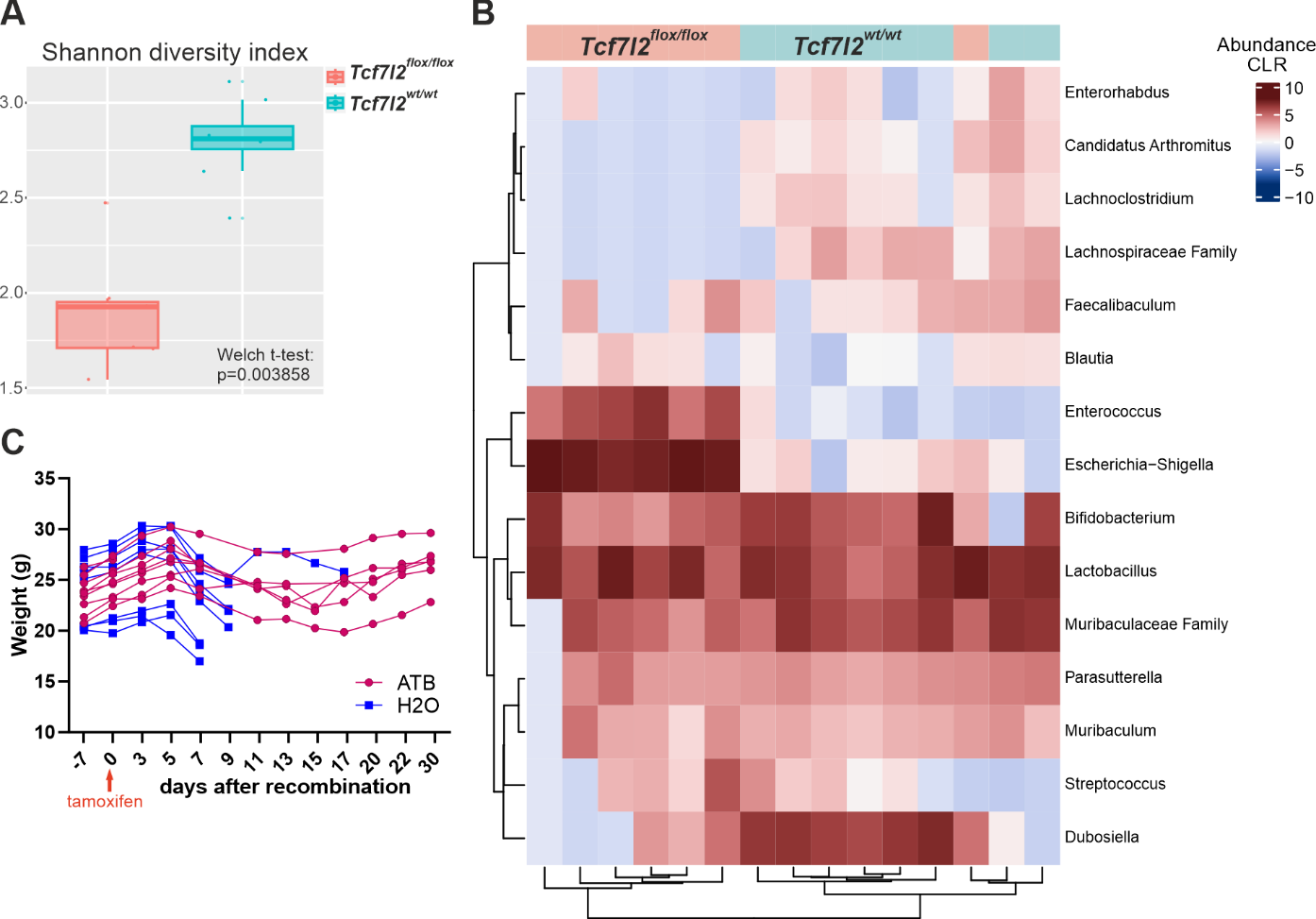
**

**Supplementary Figure S2. Analysis of the microbial composition in the small intestine of Tcf4-deficient mice.** Bacterial strains inhabiting the distal part of the small intestine of wild-type *Tcf7l2^wt/wt^/VillinCreER^T2^* (*Tcf7l2^wt/wt^*) and knockout *Tcf7l2^flox/flox^ /VillinCreER^T2^* (*Tcf7l2^flox/flox^*) mice were inspected 7 days after tamoxifen-mediated recombination.

A) A Shannon diversity index showed a significantly lower abundance of bacterial species in the distal small intestine of mice harboring conditional knockout (cKO) of the *Tcf7l2* gene.

B) A heatmap showing the distribution of the most abundant bacterial strains in the small intestine of the *Tcf7l2* WT and cKO mice. Note the increased abundance of the *Entorococcus* and *Escherichia-Shingella* strains in the Tcf4-deficient intestine. Centered log-ratio (CLR) transformed abundance of the particular bacterial species is indicated by the color scale bar.

C) Weight of the *Tcf7l2* cKO mice treated either with antibiotics or water in the survival experiment (Fig. 1E) was monitored until the death of each animal. Note the weight loss at day 7 after recombination, which is indicative of impaired intestinal epithelial function.

**
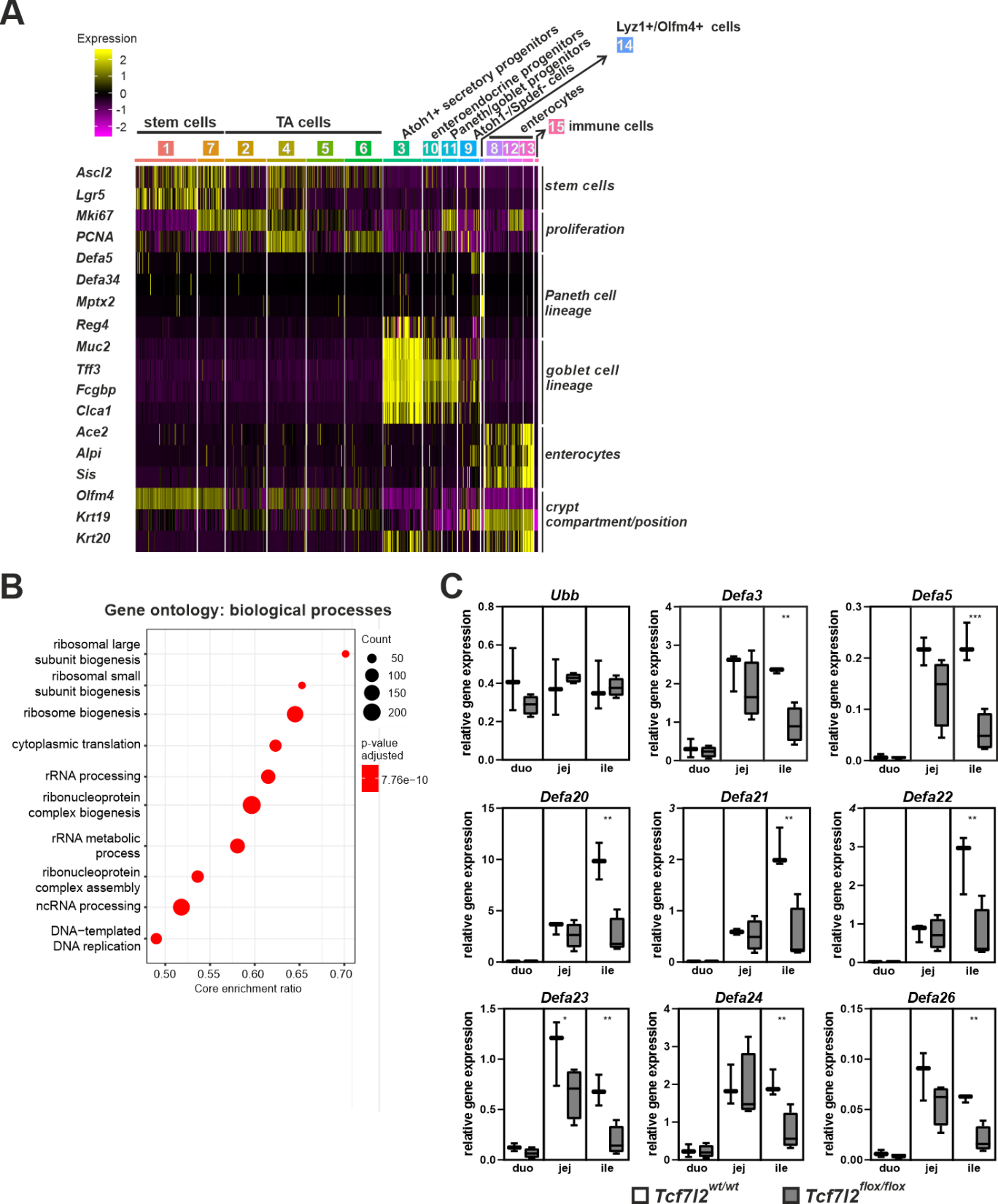
**

**Supplementary Figure S3. Single-cell (sc) and bulk RNA-seq analysis of Mki67-RFP-positive cells isolated from *Tcf7l2^wt/wt^* and *Tcf7l2^flox/flox^* mice.** Mice with the genotype *Tcf7l2^flox/flox^/Mki67^RFP/RFP^/Villin-CreER^T2^* (*Tcf7l2^flox/flox^*) were administered tamoxifen, and proliferating (i.e., Mki67-RFP-positive) epithelial cells were analyzed after 7 days. Cells from *Tcf7l2^wt/wt^/Mki67^RFP/RFP^/Villin-CreER^T2^* (*Tcf7l2^wt/wt^*) mice served as controls.

A) Heatmap from sc RNA-seq showing scaled expression of indicated marker genes and genes indicating the position of cells within the *Tcf7l2^wt/wt^* crypt compartment. Expression levels are color-coded, with yellow indicating high expression and purple indicating low expression. Ace2, angiotensin-converting enzyme 2; Alpi, alkaline phosphatase; Ascl2, achaete-scute family bHLH transcription factor 2; Clca1, chloride channel accessory 1; Defa 5/34, defensin alpha 5/34; Fcgbp, Fc gamma binding protein; Krt19/20, cytokeratin 19/20; Lgr5, leucine-rich repeat-containing G protein-coupled receptor 5; Mptx2, mucosal pentraxin 2; Muc2, mucin 2; Olfm4, olfactomedin 4; PCNA, proliferating cell nuclear antigen; Reg4, regenerating family member 4; Sis, sucrase-isomaltase; Tff3, trefoil factor 3.

B) Gene Set Enrichment Analysis (GSEA) shows the ten most altered biological processes in *Tcf7l2^flox/flox^* cells compared to *Tcf7l2^wt/wt^* cells detected using microarray expression profiling of Mki67-RFP-positive cells. Significant genes (adjusted p-value < 0.05 and |fold change (FC)| ≥ 2) were considered.

C) Decreased expression of several *Defa* genes in *Tcf7l2^flox/flox^* (n = 4) compared to *Tcf7l2^wt/wt^* (n = 3) epithelium was confirmed by RT-qPCR analysis of isolated crypts from the duodenum (duo), jejunum (jej) and ileum (ile). The phenotype is most pronounced in the distal part of the small intestine. The relative expression of the β-actin gene in the individual intestinal regions was arbitrarily set to 1; the expression of the housekeeping gene ubiquitin B (*Ubb*) is shown in the first graph. The framed areas correspond to the second and third quartiles; the median of the relative expression for each gene is shown as a black line. Statistical significance was determined using the one-way ANOVA test; *p < 0.05; **p < 0.01; ***p < 0.001.


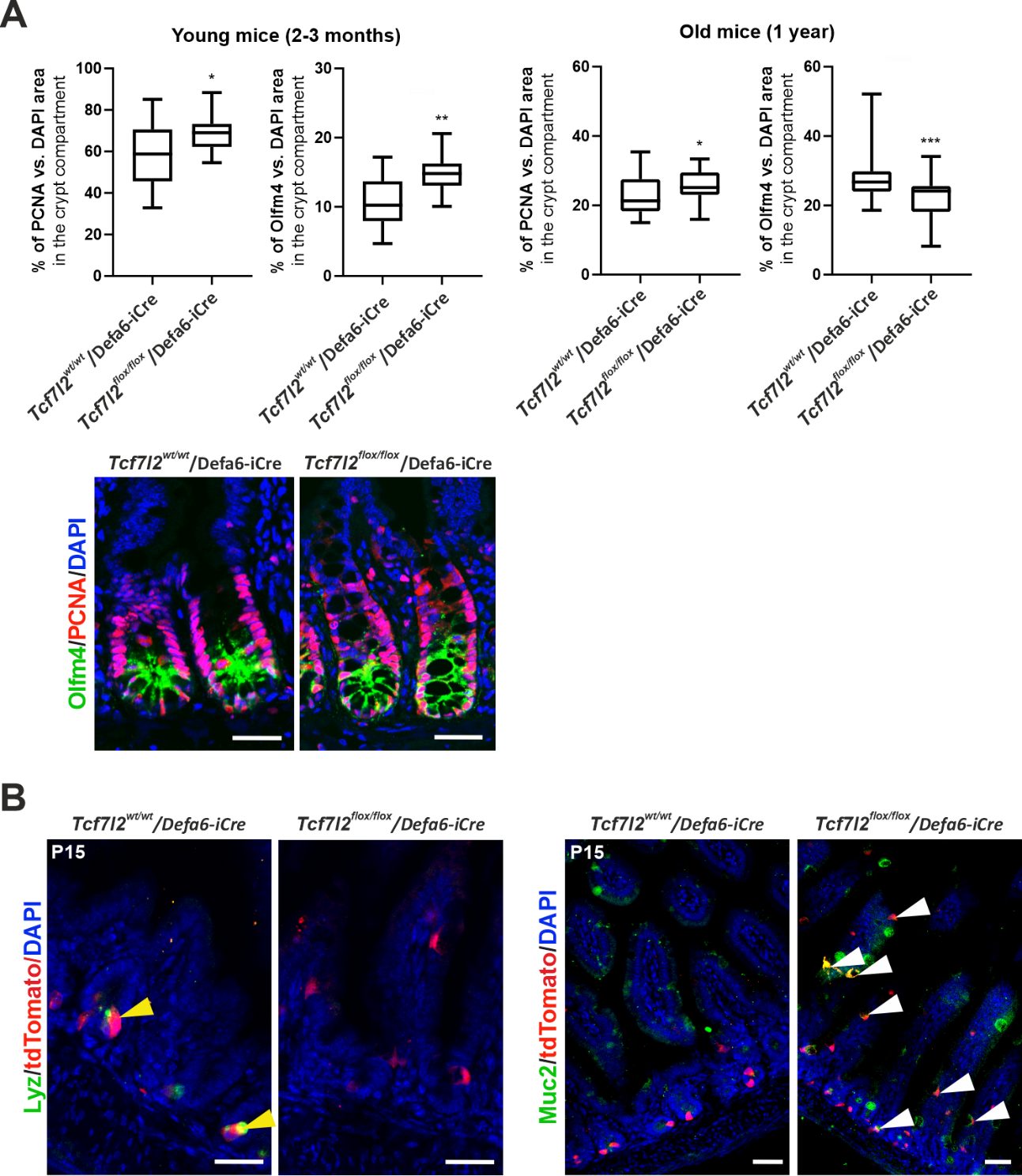


**Supplementary Figure S4. Examination of the intestinal epithelium morphology in mice carrying the homozygous conditional *Tcf7l2* allele**

A) Quantification of cells stained positive for anti-proliferating cell nuclear antigen (PCNA) and anti-olfactomedin 4 (Olfm4) in young (2-3 months) and older (1 year) mice of the wild-type (*Tcf7l2^wt/wt^*) and conditional (*Tcf7l2^flox/flox^*) *Tcf7l2* allele in Defa6-expressing cells (Defa6-iCre). For each group, at least ten microscopic fields from three biological replicates were analyzed. The signal of the antibody was normalized to the DAPI counterstain. Statistical significance was determined using the one-way ANOVA test; ^*^p < 0.05; ^**^p < 0.01; ^***^p < 0.001. Below is a representative image of immunohistochemical staining of PCNA (red nuclear signal) and Olfm4 (green signal) in a young adult mouse intestine.

B) Histological slides showing the localization of Defa-tdTom cells (tdTomato) and their colocalization with lysozyme (Lyz; yellow arrowheads) or mucin 2 (Muc2; white arrowheads) in the small intestine of indicated mouse strains 15 days after birth (P15). Animals were crossed with *Rosa26-tdTomato* reporter mice. Scale bar: 50 μm.


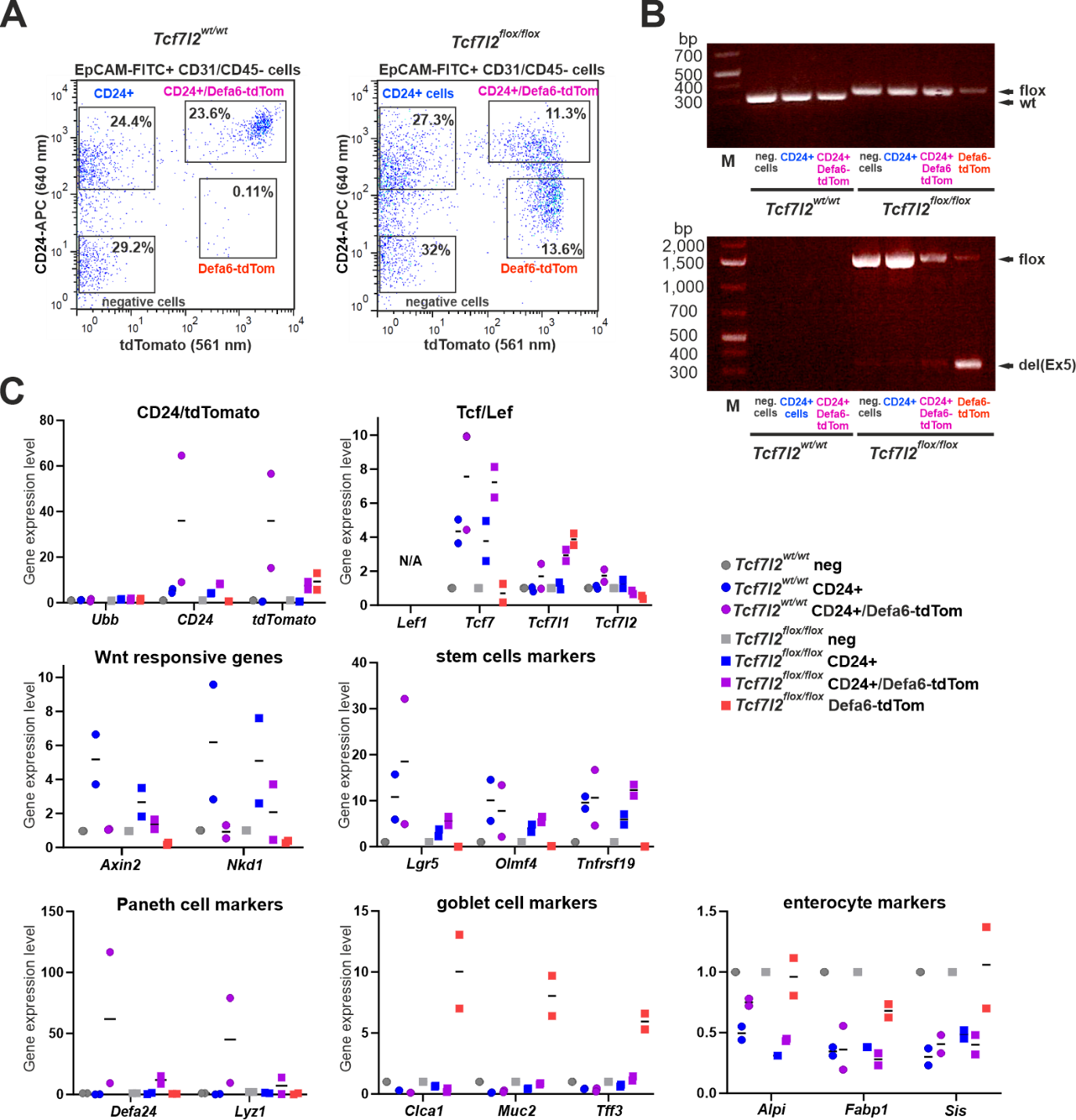


**Supplementary Figure S5. Properties of *Tcf7l2* wt (*Tcf7l2^wt/wt^*) and *Tcf7l2*-deficient (*Tcf7l2^flox/flox^*) cells isolated from the small intestine.** The mice were crossed with *Defa6-iCre/Rosa26-tdTomato* mice.

A) Representative flow cytometry plots show the gating strategy used to isolate CD24-positive and CD24-negative tdTomato^+^ (Defa6-tdTom) cells. Single cells were harvested from the middle part of the small intestine; viable (Hoechst 33258-negative), epithelial (EpCAM^+^), CD31/CD45-negative cells were sorted based on the surface marker CD24 and endogenous tdTomato fluorescence. EpCAM, epithelial cell adhesion molecule. Percentage of cells in the particular sorting gate is indicated.

B) Genotyping of the *Tcf7l2* wt and floxed (flox) alleles (top) and the recombinant Tcf7l2*^del(Ex5)^* allele [del(Ex5); bottom] was performed using DNA from all FACS-isolated cell populations. FACS, fluorescence-activated cell sorting.

C) Analysis of indicated cell type-specific genes was performed using RNA from the FACS-isolated cell populations by RT-qPCR. The relative gene expression in CD24/tdTomato-negative cells was arbitrarily set to 1. The expression of the housekeeping gene *Ubb* is shown in the first graph. The black lines indicate the mean value for two biological replicates; each reaction was performed in technical triplicates. Fabp1, fatty acid-binding protein 1; Lef1, lymphoid enhancer-binding factor 1; Nkd1, naked cuticle 1; Tnfrsf19, tumor necrosis factor receptor superfamily member 19.


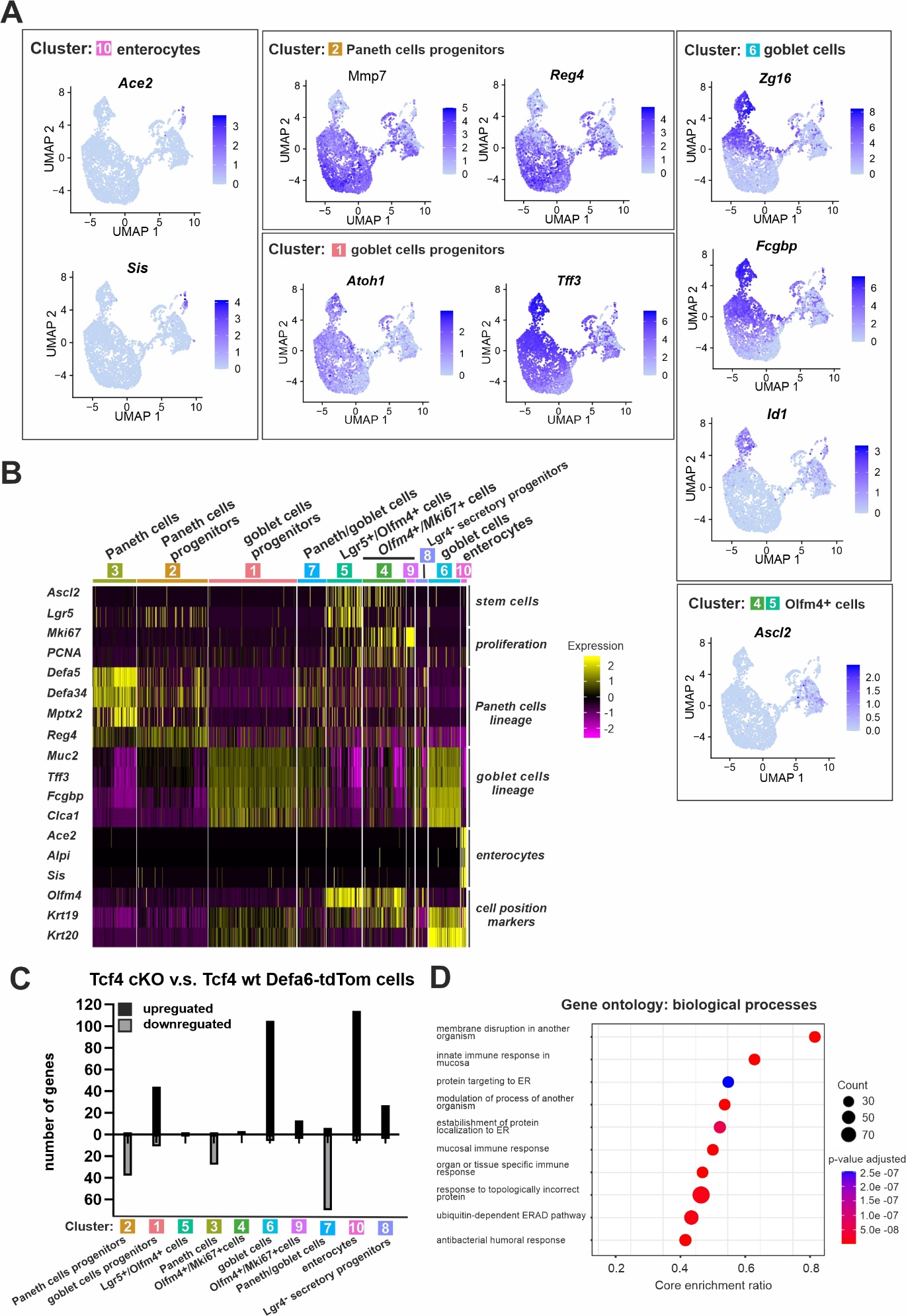


**Supplementary Figure S6. Analysis of the Tcf4 inactivation effects in Defa6-tdTom cells using scRNA and bulk RNA sequencing**

The results complement the data shown in Fig. 4.

A) UMAP plots showing the expression patterns of the different marker genes. The plots were derived from the merged datasets, i.e. from *Tcf7l2^wt/wt^* and *Tcf7l2^flox/flox^* cells. Atoh1, atonal bHLH transcription factor 1; Id1, inhibitor of DNA binding 1; Mmp7, matrix metallopeptidase 7; Zg16, zymogen granule protein 16.

B) Heatmap showing scaled expression of indicated marker genes and genes indicating the position of cells within the crypt compartment. Expression levels are color-coded, with yellow indicating high expression and purple indicating low expression.

C) Overlaps between differentially expressed genes in Defa6-tdTom cells carrying cKO alleles of *Tcf7l2* (compared to cells with wt Tcf7l2) and genes significantly enriched in cellular clusters obtained after scRNA-seq analysis of Defa6-tdTom cells isolated from the small intestinal epithelium. The values next to the selected bars indicate the percentage of genes in each cluster that belong to the differentially expressed genes obtained by bulk RNA-seq.

D) Gene Ontology (GO) biological processes enriched in *Tcf7l2*-deficient Defa6-tdTom cells when compared to *Tcf7l2* wt cells. The ten most affected pathways based on adjusted p-values are depicted. ERAD, endoplasmic reticulum-associated degradation.

**
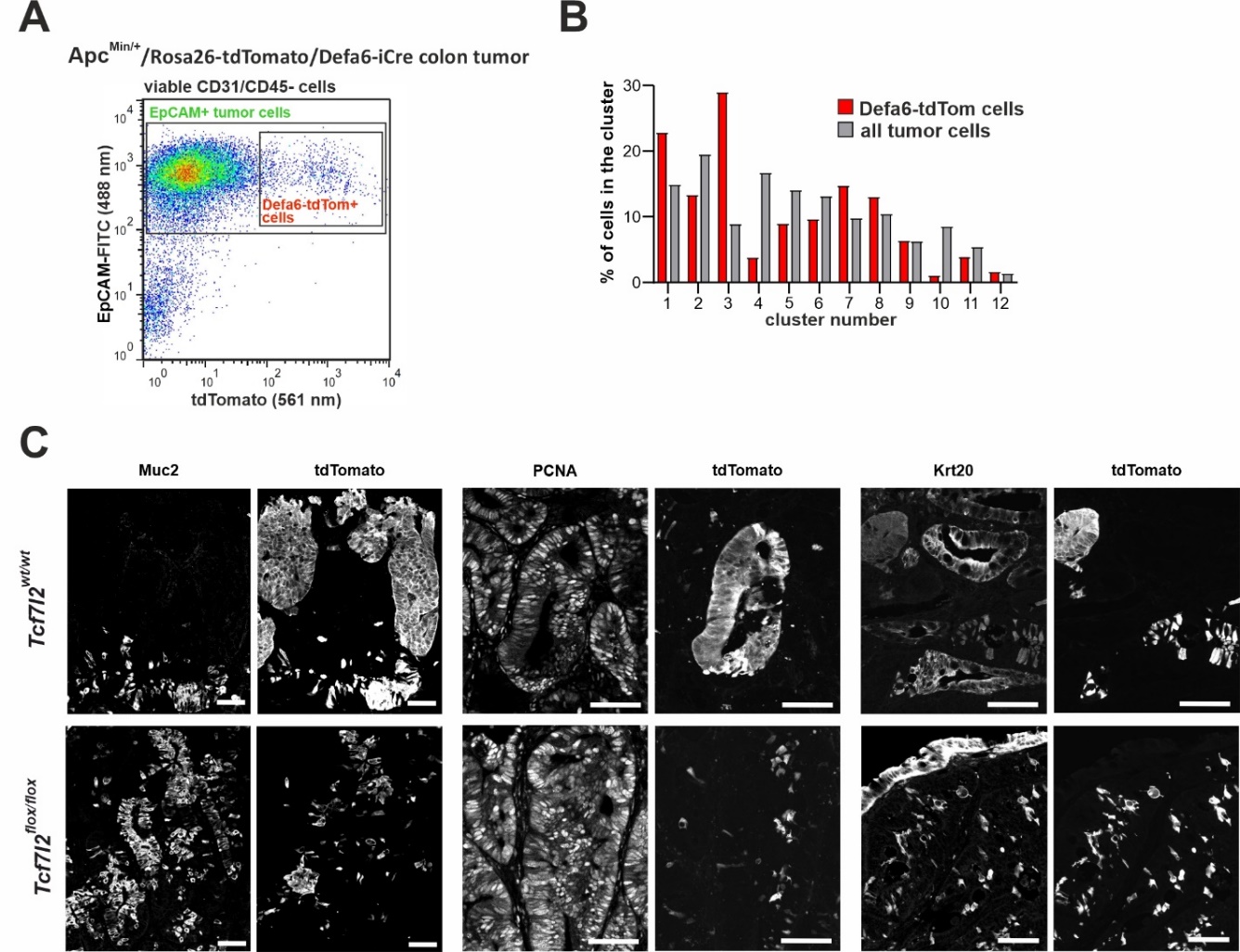
**

**Supplementary Figure S7. Sorting strategy, abundance of scRNA-seq cell clusters and immunofluorescent staining of the colon adenoma cells**

A) Representative diagram showing the fluorescence-activated cell sorting (FACS) strategy for obtaining colon tumor cells from mice with multiple intestinal neoplasia (Min) in the intestinal epithelium. Cells from dissected colonic adenomas from *Apc^Min/+^/Rosa26-tdTomato/Defa6-iCre* mice were sorted for viability (i.e., Hoechst 33258-negative cells) and EpCAM positivity; CD31-positive endothelial cells and CD45-positive leukocytes were excluded from the sorted populations. The Defa6-tdTom cells were distinguished by the red fluorescence of the tdTomato protein (Defa6-tdTom cells).

B) Bar graph showing the percentage of each cluster of Defa6-tdTom tumor cells (red) and all cells isolated from the colon tumor (gray) in Fig. 6C.

C) Grayscale version of the photomicrographs shown in Fig. 7A.

**Supplementary Table S1**

Sequences of primers used in the genotyping PCR and RT-qPCR experiments.

**Supplementary Table S2**

The genes specifically expressed in each cluster identified in Mki67-RFP-positive cells in the small intestinal crypts are listed in the corresponding sheet. Genes with the average expression |log_2_ FC| > 0.25 and p-value < 0.05 were considered as significant. FDR correction based on the total number of genes in the dataset was used to adjust the p-value. The percentage of cells with the gene expression in the first and second most significant groups is indicated.

**Supplementary Table S3**

Differentially expressed genes (DEGs) identified between Mki67-RFP-positive cells obtained from *Tcf7l2* cKO and wt small intestinal crypts. Genes with an adjusted p-value < 0.05 and |log_2_ FC | ≥ 1 were considered significant. False discovery rate (FDR) correction based on the total number of genes in the dataset was used to adjust the p-value. FC, fold change.

**Supplementary Table S4**

DEGs identified between Defa6-tdTom-positive *Tcf7l2* cKO and wt cells obtained from the ileum of the small intestine. Genes with an adjusted p-value < 0.05 and |log_2_ FC | ≥ 1 were considered significant. FDR correction based on the total number of genes in the dataset was used to adjust the p-value.

**Supplementary Table S5**

The genes specifically expressed in each cluster of Defa6-tdTom^+^ cells in the middle small intestinal crypts are listed in the corresponding sheet. Genes with the average expression |log_2_ FC| > 0.25 and p-value < 0.05 were considered as significant. FDR correction based on the total number of genes in the dataset was used to adjust the p-value. The percentage of cells with the gene expression in the first and second most significant groups is indicated.

**Supplementary Table S6**

The genes specifically expressed in each cluster identified in cells isolated from the colon tumor of *Apc^+/Min^* mice are listed in the corresponding sheet. Genes with the average expression |log_2_ FC | > 0.25 and p-value < 0.05 were considered as significant. Bonferroni correction based on the total number of genes in the dataset was used to calculate the p-value. The percentage of cells with the gene expression in the first and second most significant group is indicated.

**Supplementary Table S7**

DEGs between tdTomato-positive *Tcf7l2* cKO and wt cells obtained from the colon tumor of *Apc^+/Min^* mice. Genes with an adjusted p-value < 0.05 and |log_2_ FC | ≥ 0.5 were considered significant. FDR correction based on the total number of genes in the dataset was used to adjust the p-value. Genes characteristic of secretory precursors (pink color) and (blue color) are indicated.
